# Quality of life, bowel dysfunction, and long-term oncological outcomes after transanal *versus* laparoscopic total mesorectal excision for mid- and low- rectal cancer (Ta-LaTME study): multicentre randomized open-label trial

**DOI:** 10.1093/bjsopen/zrag045

**Published:** 2026-08-04

**Authors:** Xavier Serra-Aracil, Anna Gonzalez, Josep Bargalló, Anna Serracant, Jordi Roura, Ladislao Cayetano-Paniagua, Salvadora Delgado, Alba Zarate, Ta-LaTME Study Group, X Serra-Aracil, A Gonzalez, A Pallisera-Lloveras, A Serracant, A Garcia-Nalda, M Caraballo, E Ballesteros, J Bargalló, L Cayetano-Paniagua, S Lamas, J Roura, S Delgado, C Pericay, A Zarate

**Affiliations:** Coloproctology Unit, Parc Taulí University Hospital, Sabadell, Spain; Department of Surgery, Universitat Autònoma de Barcelona, Barcelona, Spain; General and Digestive Surgery Department, Hospital Consorcio Sanitario Terrassa, Terrassa, Spain; General and Digestive Surgery Department, Hospital Consorcio Sanitario Terrassa, Terrassa, Spain; Coloproctology Unit, Parc Taulí University Hospital, Sabadell, Spain; Department of Surgery, Universitat Autònoma de Barcelona, Barcelona, Spain; General and Digestive Surgery Department, Hospital General de Catalunya, Barcelona, Spain; General and Digestive Surgery Department, Hospital Consorcio Sanitario Terrassa, Terrassa, Spain; Coloproctology Unit, General and Digestive Surgery Department, Hospital Universitario Mutua de Terrassa, Terrassa, Spain; General and Digestive Surgery Department, Germans Trias I Pujol University Hospital, Badalona, Spain

**Keywords:** low anterior resection syndrome, patient-reported outcome measures (PROMs), bowel function, oncological safety, randomized clinical trial

## Abstract

**Background:**

Quality of life (QoL) and bowel function are key outcomes in mid- and low rectal cancer. The comparative impact of laparoscopic total mesorectal excision (LaTME) *versus* transanal total mesorectal excision (TaTME) on patient-reported outcomes and long-term oncological results in randomized trials remains unclear.

**Methods:**

This prospective multicentre randomized trial enrolled patients with resectable mid–low rectal adenocarcinoma. QoL was assessed using the 30- and 29-item European Organization for Research and Treatment of Cancer Quality of Life Core Questionnaires EORTC QLQ-C30 and QLQ-CR29, respectively) and bowel dysfunction was assessed using the Low Anterior Resection Syndrome (LARS) score before surgery and at the long-term follow-up (≥ 12 months after index surgery). The sample size calculation was based on the primary endpoint from the original randomized clinical trial (composite conversion outcome), for which 116 patients were required. The minimum oncological follow-up was 5 years. Modified intention-to-treat (mITT) and per-protocol analyses were prespecified.

**Results:**

In all, 116 patients were randomized (LaTME, 57; TaTME, 59); 105 patients were included in the mITT analysis. Sixty-seven valid QoL questionnaires were obtained (63.8%). Postoperative global health status (EORTC QLQ-C30) was high in both groups (median score 83.3) with no significant differences between them, and functional scales remained near maximum, with improved emotional functioning after LaTME only. Using the EORTC QLQ-CR29 score, TaTME showed higher postoperative stool frequency, flatulence, and faecal incontinence, with similar body image and sexual function. LARS scores (assessed only in patients without a permanent stoma) were comparable between LaTME and TaTME (median 24 *versus* 27, respectively), with major LARS (score 30–42) in 30 and 44% of LaTME and TaTME patients respectively. There were no significant differences between LaTME and TaTME in local recurrence (6.4% *versus* 3.7%, respectively), distant recurrence (14.9% *versus* 18.5%, respectively), disease-free survival, and overall survival.

**Conclusion:**

Overall QoL and bowel function remained high, with no detectable between-group differences. TaTME was associated with a higher burden of specific evacuatory symptoms on the EORTC QLQ-CR29. Long-term oncological outcomes did not differ significantly between techniques. Registration number: NCT02550769 (http://www.clinicaltrials.gov).

## Teaser

This multicentre randomized trial compared long-term quality of life (30- and 29-item EORTC Quality of Life Core Questionnaires (QLQ-C30 and QLQ-CR29, respectively)), bowel dysfunction (Low Anterior Resection Syndrome (LARS) score), and oncological outcomes after laparoscopic total mesorectal excision (LaTME) *versus* transanal total mesorectal excision (TaTME) for mid–low rectal cancer. Overall QoL remained high, with no detectable between-group differences, although TaTME showed a higher burden of specific evacuatory symptoms on the QLQ-CR29. Long-term oncological outcomes did not differ significantly between techniques, and major LARS identified the subgroup with the greatest symptom burden and QoL impairment regardless of approach.

## Introduction

Total mesorectal excision (TME), first described by Heald *et al.*^1^, remains the surgical standard for rectal cancer, enabling precise anatomical dissection and substantially reducing local recurrence. Laparoscopic TME (LaTME) was widely adopted after the COLOR II trial^2^, which demonstrated oncological equivalence with open surgery together with advantages in postoperative recovery^3^. Newer approaches, including robotic and transanal TME (TaTME), have been developed to facilitate pelvic dissection in challenging anatomy and are increasingly evaluated not only for oncological safety but also for patient-centred outcomes^4,5^.

TaTME was first described by Sylla *et al*.^6,7^. It was developed as a technical alternative for challenging pelvic dissections, facilitating sphincter preservation and distal access in patients with difficult anatomy, such as those with a narrow pelvis, male patients, or those with obesity^6,7^. The randomized Ta-LaTME^8^ and TaLaR^9^ trials have shown that TaTME significantly reduces conversion to open surgery, with postoperative morbidity and mortality comparable to LaTME. Moreover, the TaLaR trial^9^ confirmed the non-inferiority of TaTME to LaTME regarding 3-year disease-free survival (82.1% *versus* 79.4%, respectively) and demonstrated similar local recurrence rates (3.6% *versus* 4.4%, respectively), reinforcing its mid-term oncological safety despite initial concerns arising from the Norwegian moratorium^10^.

Beyond surgical and oncological outcomes, functional impact and quality of life (QoL) after TME are crucial components in the overall assessment of rectal cancer treatment. Bowel dysfunction, measured with the Low Anterior Resection Syndrome (LARS) score^11^, together with the 30- and 29-item European Organization for Research and Treatment of Cancer (EORTC) Quality of Life Core Questionnaires (QLQ-C30^12,13^ and QLQ-CR29^14,15^, respectively), allows quantification of the effect of surgery on bowel, urinary, and sexual function, as well as on patients’ global health perception. Overall QoL may remain stable despite specific symptoms due to adaptation, expectations, temporary stoma-related factors, and oncological trade-offs. Recent studies and meta-analyses^16,17^ have shown overall comparable QoL and functional outcomes between LaTME and TaTME, with no clinically relevant differences across the main EORTC domains^12,13^. However, to date, QoL and bowel dysfunction outcomes from prospective controlled randomized trials directly comparing both techniques have not been published.

The primary results of the Ta-LaTME trial^8^ have been reported previously; in this study, the prespecified secondary endpoints are reported. The Ta-LaTME trial was primarily designed to compare conversion rates to open surgery between techniques. The present study completes that trial by reporting the prespecified secondary outcomes: QoL (EORTC QLQ-C30 and QLQ-CR29), bowel dysfunction (LARS score), and long-term oncological results.

## Methods

### Trial design

This was a prospective multicentre open-label randomized trial conducted in four colorectal units in Catalonia, all with extensive experience in rectal cancer surgery. The study protocol and informed consent were approved by the Parc Taulí Ethics Committee (2014/064) and the study was registered with ClinicalTrials.gov (NCT02550769). The study followed the Declaration of Helsinki, SPIRIT 2013 and CONSORT guidelines^18^, and used the same methodology and monitoring procedures as the original Ta-LaTME protocol^19^, with follow-up exceeding 60 months.

### Participants

Adults (age ≥ 18 years) with biopsy-proven mid–low rectal adenocarcinoma staged by computed tomography and magnetic resonance imaging or endorectal ultrasound, and eligible for low anterior resection with or without chemoradiotherapy, were included. The inclusion criteria were cT1–3, cN0–1, M0 tumours ≤ 10 cm from the anal verge, medium- to low-grade histology, and American Society of Anesthesiologists (ASA) grade I–III.

Exclusion criteria included refusal to consent, tumours > 10 cm, cT4 or metastatic disease, undifferentiated adenocarcinoma, severe co-morbidity (cirrhosis, dialysis-dependent renal failure, pregnancy, ASA grade IV), body mass index < 18 or > 35 kg/m^2^, or an indication for abdominoperineal resection. Withdrawal criteria were intraoperative exclusion findings, deviation from protocol, or an inability to perform the allocated procedure. Neoadjuvant chemoradiotherapy/radiotherapy regimens followed local institutional protocols aligned with international guidelines; detailed dose and fractionation data were not captured in this patient-reported outcomes (PROMs) secondary analysis. All patients were discussed in multidisciplinary board meetings and managed accordingly, including offering adjuvant chemotherapy to patients with pN+ disease.

### Randomization and masking

Patients were randomized 1 : 1 to LaTME or TaTME using a computer-generated sequence. Patients and surgeons were unblinded, whereas follow-up was recorded by an independent assessor blinded to group assignment^8^. Central monitoring ensured data quality and protocol adherence^19^.

### Interventions and follow-up

All patients received bowel preparation, thromboembolism prophylaxis, and antibiotics per institutional standards. Procedures followed the published Ta-LaTME protocol^8,19^. LaTME consisted of laparoscopic low anterior resection^2^; TaTME was performed using a TEO platform (Karl Storz GmbH, Tuttlingen, Germany), with intracorporeal anastomosis and transanal specimen extraction^20^. Details on stapled *versus* hand-sewn anastomoses and anastomotic configuration are reported in the primary trial publication^8^. No colonic reservoir (J-pouch) reconstruction was performed.

Intraoperative and perioperative data, pathology, and 30-day morbidity were recorded. QoL (EORTC QLQ-C30^12^ and QLQ-CR29^14^) and bowel function (LARS score^11^) were assessed before treatment and at the long-term follow-up (≥ 12 months after the index surgery), generally after restoration of bowel continuity, because most patients (101 of 105) had a diverting stoma (*Fig. S1*)^19,21^. Although the original protocol planned patient-reported outcome (PRO) assessment at 6 months after ileostomy closure, this paper presents PROs collected at later long-term follow-up; the actual timing is reported in the Results. Oncological follow-up adhered to international guidelines.

### Study variables

The original primary endpoint was a composite measure of surgical conversion. Secondary endpoints included perioperative, pathological, and functional outcomes^8^.

#### Quality of life

QoL was evaluated using validated Spanish versions of the EORTC QLQ-C30^12,13^ and QLQ-CR29^14,15^, grouped into functional and symptom domains according to EORTC guidelines.

#### Bowel dysfunction

Bowel function was assessed using the LARS score^11^ in patients without a permanent stoma. This five-item questionnaire (score range 0–42) classifies LARS as no (score 0–20), minor (score 21–29), or major (score 30–42), and its components reflect patterns of incontinence and bowel frequency^22^. Patients with a permanent stoma were excluded from LARS analyses.

#### Oncological follow-up

Oncological follow-up included assessment of overall survival (OS), disease-free survival, local recurrence, and distant recurrence, with a minimum follow-up of 5 years.

### Sample size

The sample size calculation was based on the primary endpoint (composite conversion outcome). As reported previously^8^, a total of 116 patients was required. Therefore, these analyses may be underpowered and should be interpreted as exploratory.

### Monitoring

The trial was monitored by the coordinating centre (Hospital Universitari Parc Taulí). Study data were entered into electronic databases and overseen by data managers at the participating hospitals.

### Statistical analysis

Modified intention-to-treat (mITT) analyses were performed, excluding patients meeting withdrawal criteria, alongside per-protocol (PP) analyses.

EORTC QLQ-C30 and QLQ-CR29 scores were calculated according to EORTC scoring manuals and transformed to 0–100 scales^23^; questionnaires with > 30% missing items were excluded^14^. No additional item-level imputation was performed; EORTC scale analyses were conducted on an available case basis. PROM analyses were exploratory, prioritizing QLQ-C30 global health status, total/major LARS, and QLQ-CR29 stool frequency, flatulence, and faecal incontinence, with remaining scales interpreted as exploratory (no multiplicity adjustment).

Analyses were conducted using SPSS^®^ v26 (IBM, Armonk, NY, USA). Quantitative variables are expressed as the mean with standard deviation (s.d.) or as the median (first quartile, third quartile) depending on distribution; categorical variables are presented as frequencies and percentages. Within-group comparisons were made using paired *t* tests or Wilcoxon tests, whereas between-group comparisons were made using Student's *t* test or the Mann–Whitney *U* test. Categorical variables were analysed using χ^2^ or Fisher's exact tests. To identify factors associated with major LARS, binary logistic regression with forward selection was used.

Sensitivity to change in QoL was assessed using three metrics: the clinically important difference (CID)^24,25^, the standardized response mean (SRM)^26^, and Cohen's effect size (ES)^27^. CID values were interpreted as follows: 0–4 no change, 5–10 small change, 10–20 moderate change, and > 20 large change. SRM and ES were classified as negligible (0–0.2), small (0.21–0.5), moderate (0.51–0.8), and large (> 0.8)^28^.

For graphical representation, radial plots were used for QLQ-C30 and QLQ-CR29 functional and symptom scales. Symptom scales were inverted (100–score) to facilitate interpretation. Radial plots are provided as descriptive visual summaries; between-group comparisons are reported in the corresponding tables.

Oncological outcomes were analysed using Kaplan–Meier survival curves, log-rank tests, and Cox proportional hazards models to estimate hazard ratios (HRs) with their 95% confidence interval (c.i.).

*P* < 0.05 was considered statistically significant. Statistical test results are reported with corresponding 95% c.i. when applicable.

## Results

### Patient flow and inclusion

Between April 2015 and May 2021, of the 265 patients assessed for eligibility, 116 were randomized to LaTME (57) or TaTME (59). After randomization, seven patients in the LaTME group and four in the TaTME group were excluded because they did not receive the allocated procedure or because of the identification of exclusion criteria. Therefore, the mITT analysis included 50 patients in the LaTME group and 55 in the TaTME group, and the per-protocol analysis included 40 and 54 patients in the LaTME and TaTME groups, respectively (*Fig. S2*)^8,18^.

QoL questionnaires (EORTC QLQ-C30 and QLQ-CR29) and the LARS score were administered at the long-term follow-up (≥ 12 months after index surgery; *Fig. S1*). The median time from index surgery to questionnaire completion was 13 (i.q.r. 11.5-14.5) months in the LaTME group and 13 (i.q.r. 12-14) months in the TaTME group.

### Questionnaire completion

In the mITT cohort (105 patients), 67 valid questionnaires (63.8%) were available for analysis (LaTME, 68.0%; TaTME, 60.0%). Among the 77 patients alive at the time of assessment, the response rate was 87%. In the PP cohort (94 patients), 64 valid questionnaires (68%) were completed, with an 88% response rate among surviving patients (73). Although missing questionnaires were partly related to mortality/follow-up, non-response bias cannot be excluded, because patients with poorer function may have been less likely to respond. Questionnaires with > 30% missing items were excluded^14^. A comparability analysis between QoL responders and non-responders is provided in *Table S1* to assess potential non-response bias.

### EORTC QLQ-C30 questionnaires

In the mITT analysis, global health status showed high pre- and postoperative values in both groups (median > 79), with no significant differences over time or between techniques (*Table 1*). Findings were consistent in the per-protocol analysis (*Table S2*).

**Table 1:** Preoperative and postoperative EORTC QLQ-C30 scores (modified intention-to-treat population)

	LaTME (*n* = 50)					TaTME (*n* = 55)					*P*† (LaTME/TaTME)
	EORTC QLQ-CR30 score, median (Q1, Q3)	CID	ES	SRM	P* (pre/post)	EORTC QLQ-CR30 score, median (Q1, Q3)	CID TaTME	ES TaTME	SRM TaTME	P* (pre/post	
**Global health status**											
Preoperative	79.2 (56.2, 100.0)	−4.9	−0.22	−0.21	0.513	83.3 (66.7, 100.0)	7.83	0.45	0.29	0.108	0.330
Postoperative	83.3 (66.7, 93.8)					83.3 (66.7, 83.3)					0.207
**Functional scales**											
Physical functioning											
Preoperative	100.0 (86.7, 100.0)	−3.53	−0.28	−0.25	0.210	100.0 (96.7, 100.0)	1.21	0.07	0.07	0.314	0.131
Postoperative	100.0 (93.3, 100.0)					100.0 (86.7, 100.0)					0.122
Role functioning											
Preoperative	100.0 (100.0, 100.0)	2.94	0.18	0.11	0.474	100.0 (100.0, 100.0)	2.02	0.13	0.07	0.812	0.764
Postoperative	100.0 (100.0, 100.0)					100.0 (100.0, 100.0)					0.827
Emotional functioning											
Preoperative	83.3 (50.0, 100.0)	−14.22	−0.52	−0.46	0.022	83.3 (75.0, 83.3)	−8.59	−0.49	−0.32	0.052	0.934
Postoperative	100.0 (83.3, 100.0)					100.0 (83.3, 100.0)					0.541
Cognitive functioning											
Preoperative	100.0 (83.3, 100.0)	−5.88	−0.31	−0.34	0.103	100.0 (91.7, 100.0)	5.05	0.38	0.26	0.157	0.024
Postoperative	100.0 (100.0)					100.0 (75.0, 100.0)					0.110
Social functioning											
Preoperative	100.0 (83.3, 100.0)	5.39	0.24	0.17	0.314	100.0 (83.3, 100.0)	1.01	0.04	0.03	0.981	0.681
Postoperative	100.0 (66.7, 100.0)					100.0 (66.7, 100.0)					0.729
**Symptom scales**											
Fatigue											
Preoperative	22.2 (0.0, 33.3)	9.8	0.46	0.39	0.029	0.0 (0.0, 27.8)	−3.79	−0.18	−0.12	0.438	0.048
Postoperative	0.0 (0.0, 22.2)					11.1 (0.0, 27.8)					0.273
Nausea and vomiting											
Preoperative	0.0 (0.0, 0.0)	2.45	0.21	0.24	0.180	0.0 (0.0, 0.0)	−1.01	−0.21	−0.09	0.680	0.680
Postoperative	0.0 (0.0, 0.0)					0.0 (0.0, 0.0)					0.517
Pain											
Preoperative	8.3 (0.0, 33.3)	8.33	0.37	0.26	0.189	0.0 (0.0, 8.3)	−5.05	−0.38	−0.24	0.217	0.022
Postoperative	0.0 (0.0, 4.2)					0.0 (0.0, 16.7)					0.546
Dyspnoea											
Preoperative	0.0 (0.0, 8.3)	2.7	0.14	0.11	0.547	0.0 (0.0, 0.0)	−1.01	−0.1	−0.05	1.0	0.096
Postoperative	0.0 (0.0, 0.0)					0.0 (0.0, 0.0)					0.147
Insomnia											
Preoperative	0.0 (0.0, 33.3)	5.88	0.19	0.20	0.194	0.0 (0.0, 33.3)	8.08	0.24	0.19	0.374	0.812
Postoperative	0.0 (0.0, 33.3)					0.0 (0.0, 0.0)					0.342
Appetite loss											
Preoperative	0.0 (0.0, 33.3)	6.86	0.35	0.32	0.142	0.0 (0.0, 0.0)	−5.05	−0.30	−0.20	0.473	0.085
Postoperative	0.0 (0.0, 0.0)					0.0 (0.0, 16.7)					0.099
Constipation											
Preoperative	0.0 (0.0, 33.3)	0.98	0.03	0.02	0.810	0.0 (0.0, 0.0)	−14.14	−0.53	−0.32	0.077	0.037
Postoperative	0.0 (0.0, 33.3)					0.0 (0.0, 50.0)					0.850
Diarrhoea											
Preoperative	0.0 (0.0, 41.7)	−0.98	−0.03	−0.03	0.909	0.0 (0.0, 33.3)	−17.17	−0.52	−0.34	0.068	0.930
Postoperative	0.0 (0.0, 33.3)					33.3 (0.0, 83.3)					0.219
Financial difficulties											
Preoperative	0.0 (0.0, 33.3)	4.9	0.15	0.16	0.319	0.0 (0.0, 50.0)	8.08	0.23	0.23	0.152	0.810
Postoperative	0.0 (0.0, 0.0)					0.0 (0.0, 16.7)					0.776

Data are expressed as median (Q1, Q3), where Q1 and Q3 denote the first and third quartiles, respectively. LaTME, laparoscopic total mesorectal excision; TaTME, transanal total mesorectal excision; CID, clinically important difference; ES, effect size; SRM, standardized response mean. *Wilcoxon signed-rank test. †Mann–Whitney *U* test.

**Fig. 1:**
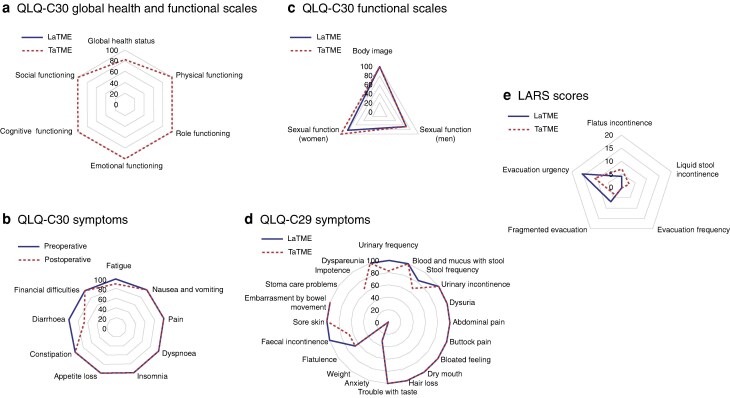
Radial plots showing changes in functional and symptom domains of the EORTC QLQ-C30 and EORTC QLQ-CR29 and LARS item scores Changes in median scores on the **a,b** EORTC QLQ-C30 and **c,d** EORTC QLQ-CR29 functional and symptom domains from before (Preop.) to after (Postop.) surgery in the LaTME and TaTME modified intention-to-treat populations. Symptom scales are presented using an inverse transformation (100–value), such that points closer to the centre indicate greater symptom burden. **e** LARS item scores; higher values reflect more severe bowel dysfunction. EORTC QLQ-CR30, 30-item European Organization for Research and Treatment of Cancer Quality of Life Core Questionnaire; EORTC QLQ-CR29, 29-item European Organization for Research and Treatment of Cancer Quality of Life Core Questionnaire; LaTME, laparoscopic total mesorectal excision; TaTME, transanal total mesorectal excision; LARS, Low Anterior Resection Syndrome.

Functional scales (physical, role, emotional, cognitive, and social functioning) remained high (median values near 100) before and after surgery in both groups (*Fig. 1a,b* and *Fig. S3*). Emotional functioning improved significantly in the LaTME group (*P* = 0.022 and *P* = 0.017 in the mITT and PP analyses, respectively), with a clinically relevant CID (∼ 10) and a small-to-moderate ES. In the TaTME group, the improvement in emotional functioning did not reach statistical significance (*P* = 0.052). Cognitive functioning was slightly lower before surgery in the TaTME group, there were no significant postoperative differences between TaTME and LaTME, except in the PP analysis, where a significant intergroup difference persisted.

Symptom scales showed a low symptom burden in both groups. Fatigue decreased significantly after LaTME (*P* = 0.029 and *P* = 0.042 in the mITT and PP analyses, respectively), with a clinically relevant CID (∼ 9–10) and moderate SRM and ES, whereas no significant change in fatigue was observed in the TaTME group. Pain decreased without reaching statistical significance, although the CID in the LaTME group was clinically meaningful (∼ 8 points). Other symptoms (nausea, dyspnoea, insomnia, appetite loss, financial difficulties) showed low or non-significant CID, ES, and SRM values, with no differences between surgical techniques.

### EORTC QLQ-CR29 questionnaires

Results for the EORTC QLQ-CR29 questionnaires administered before and after surgery are shown in *Fig. 1* and *Fig. S4*.

#### Functional scales

Body image scores remained high and stable in both groups. Sexual function in men and women was consistently low and showed no postoperative changes, with no differences between the LaTME and TaTME groups (*Table 2* and *Table S3*).

**Table 2:** Preoperative and postoperative EORTC QLQ-CR29 scores (modified intention-to-treat population)

	LaTME (*n* = 50)					TaTME (*n* = 55)					*P*† (LaTME/TaTME)
	EORTC QLQ-CR29 score, median (Q1, Q3)	CID	ES	SRM	P* (pre/post)	EORTC QLQ-CR29 score, median (Q1, Q3)	CID	ES	SRM	P* (pre/post)	
**Functional scales**											
Body image											
Preoperative	100 (75, 100)	−3.59	−0.23	−0.13	0.180	100 (88.9, 100)	1.16	−0.13	−0.13	0.548	0.230
Postoperative	100 (100, 100)					100 (88.9, 100)					0.557
Sexual function											
Men											
Preoperative	66.7 (66.7, 100)	1.67	0.12	0.12	0.463	66.7 (66.7, 100)	0.5	0.07	0.05	0.618	0.688
Postoperative	66.7 (33.3, 100)					66.7 (66.7, 100)					0.465
Women											
Preoperative	100 (66.7, 100)	1.85	0.17	0.20	1.000	66.7 (66.7, 100)	−19.05	−0.83	−1.07	0.059	0.252
Postoperative	83.3 (66.7, 100)					100 (66.7, 100)					0.475
**Symptom scales**											
Urinary frequency											
Preoperative	33.3 (0, 54.2)	18.14	0.61	0.46	0.014	0 (0, 50)	2.02	0.07	0.05	0.786	0.082
Postoperative	0 (0, 33.3)					16.7 (0, 33.3)					0.341
Blood and mucus with stool											
Preoperative	16.7 (0, 50)	27.45	0.93	0.96	0.001	33.3 (0, 50)	23.74	0.85	0.75	0.001	0.678
Postoperative	0 (0, 0)					0 (0, 0)					0.109
Stool frequency											
Preoperative	16.7 (0, 33.3)	−4.08	−0.21	−0.13	0.675	8.3 (0, 16.7)	−21.43	−0.88	−0.52	0.017	0.372
Postoperative	16.7 (0, 33.3)					33.3 (16.7, 62.5)					0.027
Urinary incontinence											
Preoperative	0 (0, 0)	−0.98	−0.05	−0.04	0.862	0 (0, 0)	−8.08	−0.42	−0.39	0.029	0.697
Postoperative	0 (0, 0)					0 (0, 33.3)					0.138
Dysuria											
Preoperative	0 (0, 0)	−0.98	−0.12	−0.08	0.655	0 (0, 0)	−5.05	–	−0.27	0.102	0.160
Postoperative	0 (0, 0)					0 (0, 0)					0.939
Abdominal pain											
Preoperative	0 (0, 33.3)	8.82	0.32	0.27	0.146	0 (0, 33.3)	3.03	0.11	0.09	0.557	0.242
Postoperative	0 (0, 33.3)					0 (0, 16.7)					0.902
Buttock pain											
Preoperative	0 (0, 33.3)	0.98	0.04	0.03	0.968	0 (0, 0)	−9.09	−0.62	−0.27	0.146	0.048
Postoperative	0 (0, 33.3)					0 (0, 33.3)					0.886
Bloated feeling											
Preoperative	0 (0, 33.3)	8.82	0.35	0.29	0.097	0 (0, 33.3)	1.01	0.03	0.03	0.936	0.957
Postoperative	0 (0, 0)					0 (0, 33.3)					0.255
Dry mouth											
Preoperative	0 (0, 41.7)	13.73	0.40	0.39	0.038	0 (0, 33.3)	−3.03	−0.11	−0.08	0.675	0.311
Postoperative	0 (0, 33.3)					0 (0, 33.3)					0.301
Hair loss											
Preoperative	0 (0, 0)	1.96	0.07	0.06	0.762	0 (0, 33.3)	6.06	0.24	0.25	0.403	0.193
Postoperative	0 (0, 0)					0 (0, 0)					0.960
Trouble with taste											
Preoperative	0 (0, 8.3)	8.82	0.32	0.27	0.162	0 (0, 16.7)	3.03	0.09	0.08	0.776	0.847
Postoperative	0 (0, 0)					0 (0, 0)					0.194
Anxiety											
Preoperative	66.7 (33.3, 100)	−19.61	−0.57	−0.50	0.005	66.7 (33.3, 100)	−10.23	−0.38	−0.29	0.277	0.293
Postoperative	66.7 (66.7, 100)					66.7 (66.7, 100)					0.887
Weight											
Preoperative	100 (66.7, 100)	−6.86	−0.24	−0.20	0.243	100 (66.7, 100)	−1.92	−0.08	−0.07	0.892	0.758
Postoperative	100 (66.7, 100)					100 (10, 100)					0.630
Flatulence											
Preoperative	16.7 (0, 66.7)	−3.72	−0.11	−0.08	0.965	0 (0, 33.3)	−27.08	−0.89	−0.6	0.008	0.150
Postoperative	33.3 (0, 33.3)					33.3 (0, 66.7)					0.145
Faecal incontinence											
Preoperative	0 (0, 33.3)	−0.85	−0.13	−0.08	0.636	0 (0, 0)	−29.91	−1.03	−0.77	0.002	0.061
Postoperative	0 (0, 33.3)					33.3 (0, 66.7)					0.140
Sore skin											
Preoperative	0 (0, 33.3)	−7.52	−0.49	−0.27	0.153	0 (0, 0)	−28.87	−2.08	−0.61	0.006	0.020
Postoperative	0 (0, 33.3)					0 (0, 66.7)					0.332
Embarrassment by bowel movement											
Preoperative	0 (0, 8.3)	−6.8	−0.45	−0.20	0.359	0 (0, 0)	−13.10	−0.69	−0.53	0.013	0.039
Postoperative	0 (0, 33.3)					0 (0, 33.3)					0.450
Stoma care problems											
Preoperative	–	–	–	–	–	–	–	–	–	–	–
Postoperative	–					–					–
Impotence											
Preoperative	33.3 (0, 66.7)	0	0	0	0.971	0 (0, 16.7)	−21.95	−0.67	−0.67	0.011	0.047
Postoperative	33.3 (0, 58.3)					33.3 (0, 66.7)					0.918
Dyspareunia											
Preoperative	0 (0, 33.3)	−3.81	−0.68	−0.41	0.317	0 (0, 66.7)	14.29	0.34	0.38	0.317	0.805
Postoperative	0 (0, 33.3)					0 (0, 0)					0.669

Data are expressed as median (Q1, Q3), where Q1 and Q3 denote the first and third quartiles, respectively. LaTME, laparoscopic total mesorectal excision; TaTME, transanal total mesorectal excision; CID, clinically important difference; ES, effect size; SRM, standardized response mean. *Wilcoxon signed-rank test; †independent-samples Mann–Whitney *U* test.

#### Symptom scales

Both groups showed a marked reduction in blood or mucus in the stool after surgery (*P* < 0.001). Urinary frequency improved after LaTME (*P* = 0.014), whereas urinary incontinence increased after TaTME (*P* = 0.029), without significant between-group differences.

TaTME patients reported higher postoperative stool frequency (*P* = 0.017 and *P* = 0.027 in the mITT and PP analyses, respectively), along with increased flatulence and faecal incontinence: *P* = 0.008 (mITT) and *P* = 0.002 (PP), respectively; *Table 2* and *Table S3*). Weight remained stable. Anxiety decreased significantly after surgery in the LaTME group (*P* = 0.005), with no meaningful changes in the TaTME group. These findings indicate a higher burden of specific evacuatory symptoms after TaTME, which is relevant for clinical interpretation and patient counselling.

### LARS scores

#### Recruitment and follow-up

In the mITT analysis (105 patients), valid LARS questionnaires were obtained from 33 of 50 LaTME patients (66%) and 32 of 55 TaTME patients (58%), with an effective response rate of 87% among patients alive at the time of assessment (LaTME, 94%; TaTME, 81%). Postoperative major LARS occurred in 10 of 33 LaTME patients (30%) *versus* 14 of 32 TaTME patients (44%) in the mITT cohort, and in 9 of 30 LaTME patients (30%) *versus* 14 of 32 TaTME patients (44%) in the PP cohort (*Table 3*). A comparability analysis between LARS responders and non-responders is provided in *Table S4* to assess potential non-response bias. Preoperative/postoperative analyses and graphical LARS severity distribution are shown in *Table S5*, *Fig. 1*, and *Fig. S5*, respectively.

**Table 3:** Postoperative LARS scores for the LaTME and TaTME groups in the mITT and PP populations

	mITT analysis			PP analysis		
	LaTME (*n* = 33 of 50, 66%)	TaTME (*n* = 32 of 55, 58.2%)	P*	LaTME (*n* = 30 of 40, 75%)	TaTME (*n* = 32 of 54, 59.3%)	P*
No LARS	12 (36.4%)	12 (37.5%)	0.346	10 (33.3%)	12 (37.5%)	0.262
Minor LARS	11 (33.3%)	6 (18.8%)		11 (36.7%)	6 (18.8%)	
Major LARS	10 (30.3%)	14 (43.8%)		9 (30%)	14 (43.8%)	
**No/minor LARS *versus* major LARS**						
No/minor LARS	23 (69.7%)	18 (56.3%)	0.310†	21 (70%)	18 (56.3%)	0.302†
Major LARS	10 (30.3%)	14 (43.8%)		9 (30%)	14 (43.8%)	
**No LARS *versus* minor/major LARS**						
No LARS	12 (36.4%)	12 (37.5%)	0.999†	10 (33.3%)	12 (37.5%)	0.795†
Minor/major LARS	21 (63.6%)	20 (62.5%)		20 (66.7%)	20 (62.5%)	
**Pattern type**						
Incontinence-dominant pattern (%), median (Q1, Q3)	40 (0, 70)	100.0 (32.5, 100.0)	0.012‡	40 (0, 70)	100.0 (32.5, 100.0)	0.011‡
Frequency-dominant pattern (%), median (Q1, Q3)	62.5 (34.4, 78.1)	56.3 (34.4, 78.1)	0.630‡	62.5 (34.8, 78.1)	56.3 (34.8, 78.1)	0.483‡

Values are *n* (%) unless otherwise stated. LARS scores were used to divide patients into three groups: no LARS (score 0–20), minor LARS (score 21–29), and major LARS (score 30–42). LARS, Low Anterior Resection Syndrome; LaTME, laparoscopic total mesorectal excision; TaTME, transanal total mesorectal excision; mITT, modified intention-to-treat; PP, per-protocol. *Pearson χ^2^ test, except †Fisher's exact test and ‡independent-samples Mann–Whitney *U* test.

In the PP analysis (94 patients), valid LARS questionnaires were available for 30 of 40 patients in the LaTME group (75%) and 32 of 54 patients in the TaTME group (59%), with a response rate of 88% among surviving patients (*Table S6*).

#### Between-group comparisons (mITT and PP analyses)

Global LARS scores and individual items are presented in *Table 3* and Tables  *S5* and *S6*, with severity distributions shown in *Fig. 1* and *Fig. S5*. In the mITT analysis, median LARS scores were similar between the LaTME and TaTME groups (24 (i.q.r. 14.5-33.5) *versus* 27 (i.q.r. 16.5-37.5), respectively; *P* = 0.450; *Table S5*), findings replicated in the PP analysis (median 24 (i.q.r. 14.5-33.5) *versus* 27.5 (i.q.r. 17-38); *P* = 0.450, respectively; *Table S6*). The proportion of major LARS was also comparable between the LaTME and TaTME groups (mITT: 30.3% *versus* 43.8%, respectively, in the mITT analysis (*P* = 0.350); 30% *versus* 43.8%, respectively, in the PP analysis (*P* = 0.260); *Table 3*).

In preoperative and postoperative analyses (*Tables S5, S6*), TaTME showed significant increases in liquid stool incontinence and urgency (both *P* = 0.001), whereas LaTME changes were limited to increased fragmented evacuation (*P* = 0.002). The postoperative bowel movement frequency (LARS item) was similar between groups. This differs from the QLQ-CR29 symptom scale, where stool frequency was higher after TaTME.

#### Univariable analysis

Several postoperative morbidity indicators were associated with major LARS in univariable analyses (overall morbidity, Clavien–Dindo grade > II complications, and Comprehensive Complication Index (CCI)^29^; *Table S7*). Given the limited number of events and collinearity between morbidity variables, no multivariable model was fitted. Major LARS was more frequent in patients with than without postoperative morbidity (62.5% *versus* 34.1%; *P* = 0.039) and in those with Clavien–Dindo grade > II complications (*P* = 0.046). No significant associations were found with age, sex, body mass index, tumour height, surgical approach (LaTME *versus* TaTME), or neoadjuvant therapy.

#### Association between major LARS and post-treatment QoL

As shown in *Table S7*, patients with major LARS had significantly lower scores across multiple EORTC QLQ-C30 domains, including global health status (*P* < 0.001), physical functioning (*P* = 0.007), role functioning (*P* = 0.008), and social functioning (*P* = 0.025), together with higher levels of fatigue, insomnia, and diarrhoea (*P* ≤ 0.004). Similarly, in the EORTC QLQ-CR29 domains, major LARS was associated with increased stool frequency, flatulence, and faecal incontinence (all *P* < 0.001), as well as symptoms related to skin irritation or embarrassment when defaecating (*P* ≤ 0.013).

### Long-term oncological outcomes

Long-term oncological outcomes were analysed in 101 patients (LaTME, 47; TaTME, 54) with available oncological follow-up data. Four patients from the mITT cohort were excluded from this analysis due to missing long-term follow-up data.

**Fig. 2:**
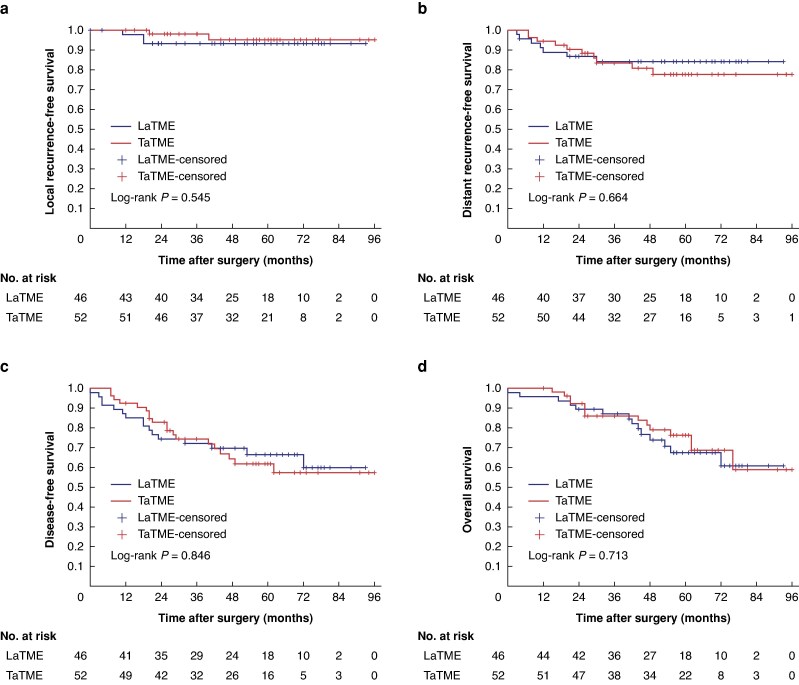
Kaplan–Meier curves showing long-term oncological outcomes by surgical technique (LaTME *versus* TaTME) **a** Local recurrence-free survival; **b** distant recurrence-free survival; **c** disease-free survival; **d** overall survival. LaTME, laparoscopic total mesorectal excision; TaTME, transanal total mesorectal excision.

With a median follow-up of 55 months, no significant differences were observed between LaTME and TaTME for any oncological endpoint (*Fig. 2*). No differences were detected in long-term oncological outcomes between techniques; however, the confidence intervals were wide.

Local recurrence occurred in 5 of 101 patients (5.0%), with rates of 6.4% (3 of 44) in the LaTME group and 3.7% (2 of 54) in the TaTME group. Kaplan–Meier curves for local recurrence (*Fig. 2a*) overlapped, with no statistically significant difference (log-rank *P* = 0.545; HR 1.72 (95% c.i. 0.28 to 10.32; *P* = 0.551)).

Distant recurrence was recorded in 7 of 47 patients (14.9%) after LaTME and in 10 of 54 patients (18.5%) after TaTME, without significant differences (log-rank *P* = 0.664; HR 1.07 (95% c.i. 0.45 to 2.58; *P* = 0.875)). Disease-free survival was similar between groups (*Fig. 2c*; log-rank *P* = 0.846; HR 1.01 (95% c.i. 0.53 to 1.93; *P* = 0.971)), as was overall survival (*Fig. 2d*; log-rank *P* = 0.713; HR 1.30 (95% c.i. 0.64 to 2.63; *P* = 0.471)).

## Discussion

Available evidence on functional outcomes after TaTME comes mainly from cohort studies and meta-analyses, showing QoL and bowel function broadly comparable to LaTME^16,17^. However, randomized trials directly assessing PROs are lacking. In this context, the present analysis provides the first randomized multicentre data comparing TaTME to LaTME for QoL, bowel dysfunction, and long-term oncological results, offering a robust view of the overall impact of both techniques.

In analysing the EORTC QLQ-C30 and QLQ-CR29 questionnaires, three clinically oriented metrics were combined into CID^24,25^, SRM^26^, and ES^27,28^, in addition to conventional statistical testing. This approach is particularly valuable in QoL research because it helps distinguish clinically meaningful changes from statistically non-relevant variation, both within and between groups.

In the present study, global quality of life (EORTC QLQ-C30) remained high and stable in both groups, with no detectable differences between TaTME and LaTME, consistent with comparative studies and meta-analyses reporting no clinically relevant differences in overall QoL^16,17^. Given the high EORTC QLQ-C30 scores at baseline and follow-up in long-term survivors, a ceiling effect cannot be excluded. Changes observed in emotional functioning and fatigue should be interpreted as exploratory signals rather than conclusive between-technique differences. Therefore, this apparent similarity should not be overinterpreted as equivalence between techniques.

Functional scales (physical, role, emotional, cognitive, and social functioning) showed scores close to 100 before and after surgery, consistent with prospective series describing near-complete functional recovery regardless of surgical approach^30,31^. The improvement in emotional functioning seen after LaTME, although statistically significant, has not been reported consistently elsewhere and likely reflects random variation rather than a technique-specific effect^32^. Similarly, the small preoperative difference in cognitive functioning in the TaTME group has not been described in previous studies.

Regarding symptom scales, both techniques showed low symptom burden, in line with meta-analyses reporting no meaningful differences^16^. The significant reduction in fatigue after LaTME has not been identified as a differential pattern, because most studies describe similar improvements with both approaches^17,32^. The clinically relevant, although non-significant, reduction in pain is comparable to previously published values for both TaTME and LaTME^30,31^.

The EORTC QLQ-CR29 results showed that body image scores remained high and stable in both groups. Sexual function in men and women remained low and unchanged after surgery, consistent with studies reporting no significant differences between TaTME and LaTME^16,17^. The literature suggests that TME may impair sexual function regardless of surgical approach, with no clear advantage demonstrated for TaTME^30,31^. Regarding urinary symptoms, urinary frequency was improved after LaTME and there was increased urinary incontinence after TaTME, although without between-group differences; these findings align with reports of similar urinary profiles and no clinically relevant differences^17^. The marked reduction in blood and mucus in stool reflects resolution of tumour-related symptoms rather than an effect of surgical technique. Conversely, the increase in stool frequency, flatulence, and faecal incontinence after TaTME mirrors studies suggesting greater evacuatory disturbance following the transanal approach, particularly for low tumours^32^, although other reports have not confirmed this difference^16^. Finally, the significant reduction in anxiety after LaTME, not observed after TaTME, is consistent with previous studies describing postoperative improvements in emotional wellbeing^16,30^. Overall QoL was high and showed no detectable differences; however, TaTME was associated with a higher burden of specific evacuatory symptoms. This apparent dissociation between evacuatory symptoms and stable global QoL may reflect ceiling effects of global QoL scales and/or adaptation (response shift) in long-term survivors. Importantly, subgroup analysis in the present study showed that patients with major LARS have markedly worse QoL and a higher symptom burden, suggesting that the clinical relevance of these symptoms is concentrated in this subgroup.

In this randomized trial, global LARS scores and severity distribution were comparable between the TaTME and LaTME groups, with no significant differences in median scores or in the proportion of major LARS. These findings are consistent with meta-analyses and prospective studies reporting similar rates of major LARS and mid-term evacuatory function regardless of surgical approach^16,17,30,31^.

In the within-group analyses, the TaTME group had significant increases in urgency and liquid-stool incontinence, whereas changes after LaTME were more modest. Previous studies have suggested that the transanal dissection may more markedly affect rectal sensitivity, particularly in low tumours^32^, although other reports have not reproduced this difference, leaving its clinical relevance uncertain^16^.

The observed association between major LARS and poorer QoL across multiple QLQ-C30 and QLQ-CR29 domains is consistent with previous evidence demonstrating a strong link between severe bowel dysfunction and impaired physical, emotional, and social wellbeing in rectal cancer survivors^17^. These findings support tailored preoperative counselling and proactive postoperative management (for example, structured follow-up and pelvic floor/bowel rehabilitation), particularly in patients who develop major LARS and/or postoperative morbidity. Postoperative morbidity (Comprehensive Complication Index (CCI))^29^ was associated with major LARS, suggesting that the prevention of complications may help mitigate long-term bowel dysfunction. Overall, the key clinical message is not only the comparison between techniques but the identification of major LARS as the subgroup with the greatest symptom burden and impaired QoL.

With a median follow-up of 55 months, no significant differences were observed in long-term oncological outcomes between techniques, although confidence intervals were wide. These findings extend the earlier Ta-LaTME results, which had already shown low and comparable local recurrence rates at 39 months^8^, with no clustering of pelvic failures in TaTME. The present data confirm that this pattern persists beyond 5 years. The results are also consistent with the TaLaR trial^9^, which demonstrated the oncological non-inferiority of transanal TME versus laparoscopic TME, with similar 3-year disease-free survival (82.1% *versus* 79.4%, respectively) and local recurrence rates (3.6% *versus* 4.4%, respectively). Together, and in contrast to the concerns raised by the Norwegian moratorium^10^, these results suggest that, when performed by experienced teams with a standardized technique and an adequate learning curve, TaTME provides mid- and long-term oncological outcomes comparable to LaTME.

Among the limitations of this study, the sample size was powered for the original primary endpoint (surgical conversion) rather than for detecting differences in QoL or bowel dysfunction; thus, the functional analyses may be underpowered. Accordingly, the absence of statistically significant differences in PROMs should not be interpreted as evidence of equivalence between techniques. Although questionnaire response rates were high among surviving patients (87%), only 63.8% of the mITT cohort provided valid questionnaires; therefore, selection bias towards better-recovered responders cannot be excluded. In addition, the exclusion of patients with a permanent stoma from the LARS analysis reduces the effective sample size and limits the generalizability of these findings to the entire TME population. Comparability analyses showed differences between responders and non-responders (*Tables S1, S4*); therefore, the magnitude and direction of non-response bias cannot be fully determined.

## Supplementary Material

zrag045_Supplementary_Data

## Data Availability

Deidentified participant data will be shared upon reasonable request to the corresponding author (subject to institutional approvals).

## Collaborators

Members of the Ta-LaTME Study Group are: X. Serra-Aracil, A. Gonzalez, A. Pallisera-Lloveras, A. Serracant, A. Garcia-Nalda, M. Caraballo, E. Ballesteros (Parc Taulí University Hospital, Sabadell, Spain); J. Bargalló, L. Cayetano-Paniagua, S. Lamas (Hospital Consorcio Sanitario Terrassa, Terrassa, Spain); J. Roura (Hospital General de Catalunya, Barcelona, Spain); S. Delgado, C. Pericay (Mutua de Terrassa University Hospital, Terrassa, Spain); and A. Zarate (Germans Trias i Pujol University Hospital, Badalona, Spain).
