# Supplementary material for: Quality of life, bowel dysfunction, and long-term oncological outcomes after transanal versus laparoscopic total mesorectal excision for mid- and low- rectal cancer (Ta-LaTME study): multicentre randomized open-label trial
Source: BJS Open. 2026 Aug 4;10(4):zrag045. doi: 10.1093/bjsopen/zrag045 (PMC13436816; doi:10.1093/bjsopen/zrag045)
Supplement: Supplementary file 1 [file zrag045_supplementary_data.zip › 2018- IJCD-Protocolo TaTME-LLAR.pdf]

International Journal of Colorectal Disease
https://doi.org/10.1007/s00384-018-2996-8

CLINICAL STUDY PROTOCOL

Study protocol for a multicenter prospective controlled and randomized trial of transanal total mesorectal excision versus laparoscopic low anterior resection in rectal cancer

X. Serra-Aracil[1] · Alba Zarate[1] · L. Mora[1] · S. Serra-Pla[1] · A. Pallisera[1] · J. Bonfill[2] · J. Bargalló[3] · A. Pando[4] · S. Delgado[4] · E. Balleteros[5] · C. Pericay[6]

Accepted: 26 February 2018
© Springer-Verlag GmbH Germany, part of Springer Nature 2018

FOR APPROVAL

Abstract

Purpose Compared with the open approach, laparoscopic total mesorectal excision (TME) achieves faster patient recovery, reduces morbidity rates, and shortens hospital stay. However, in laparoscopic low anterior resection (L-LAR), conversion to open surgery is required in almost 20% of cases. Transanal TME (Ta-TME) combined with laparoscopy, also called hybrid natural *orifice* transluminal endoscopic surgery (NOTES), is a less invasive procedure that can overcome some of the limitations of laparoscopic rectal surgery. In this study, we aim to determine whether Ta-TME has a lower rate of conversion to open surgery than L-LAR, and thus achieves faster patient recovery without altering the pathological, functional, or oncological results. The main objective is to compare the results for conversion to open surgery between Ta-TME and L-LAR.
Methods Multicenter, prospective randomized controlled study of patients diagnosed with rectal adenocarcinoma who will be randomly allocated to Ta-TME or L-LAR groups after the application of inclusion and exclusion criteria. The main endpoint is conversion to open surgery and the secondary endpoints are general morbidity and mortality and hospital stay. Demographic, surgical, and pathological variables will also be studied, along with quality of life and survival. A sample size of 53 patients per group is calculated. With an estimated loss of 10%, the final sample required will be 116 patients.
Conclusions Ta-TME achieves a lower conversion rate to open surgery than L-LAR, thus improving patient recovery and reducing overall morbidity.
Trial registration ClinicalTrials.gov Identifier: NCT02550769. Registration no. Ethical and Clinical Research Committee, Parc Taulí University Hospital: ID 2014/064.

Keywords Rectal cancer · Laparoscopic low anterior resection · Total mesorectal excision · TME Transanal total mesorectal excision · Ta-TME · Rectal hybrid NOTES

✉ Alba Zarate
azaratepi@gmail.com

1 Coloproctology Unit, General and Digestive Surgery Department, Parc Tauli University Hospital, Universitat Autonoma de Barcelona, Parc Tauli s/n. 08208, Sabadell, Barcelona, Spain

2 General and Digestive Surgery Department, Hospital General de Catalunya, Barcelona, Spain

3 General and Digestive Surgery Department, Hospital de Terrassa, Terrassa, Spain

4 Coloproctology Unit, General and Digestive Surgery Department, Hospital Universitario Mutua de Terrassa, Terrassa, Spain

5 Radiology Department, Parc Tauli University Hospital, Universitat Autonoma de Barcelona, Sabadell, Barcelona, Spain

6 Medical Oncology Department, Parc Tauli University Hospital, Universitat Autonoma de Barcelona, Sabadell, Barcelona, Spain

Background

Minimally invasive techniques are known to present advantages over conventional surgery in terms of patient recovery. In rectal cancer surgery, the advantages of laparoscopic surgery are also well established; the laparoscopic approach reduces hospital stay and shortens recovery time while achieving the same oncological results as total mesorectal excision (TME) described by Heald [1]. The main limitation of laparoscopic rectal surgery is the high incidence of conversion to open surgery which, although the ROLARR trial [2] reported rates of 8–12%, is close to 20% in most series [3] .

Since the first description of transanal endoscopic microsurgery (TEM) for the resection of rectal tumors by Buess in

1984 [4], the technique has evolved considerably and is currently applied in other rectal and pelvic pathologies which have traditionally been approached via laparotomy. In recent years, transanal endoscopic surgery via natural orifice translumenal endoscopic surgery (NOTES) has been used to access the peritoneal cavity and to perform intra-abdominal procedures. With the experience acquired by certain groups in transanal endoscopic surgery and thanks to the demonstration of the advantages of NOTES [5–7], the use of transanal techniques for oncological surgery of the rectum is becoming more frequent.

Pure NOTES [8], in which TME is performed excusively through the transanal approach, presents limitations and substantial technical difficulties. However, "hybrid" NOTES [9–12] which combines laparoscopic and transanal endoscopic surgery can carry out the most critical steps of abdominal surgery safely through laparoscopy and facilitate the pelvic dissection of the rectum thanks to the use of TEM.

Transanal total mesorectal excision (Ta-TME) was first described by Sylla [13], using TEM equipment. In recent years, the use of the single port as a transanal approach has become widespread. At our center, after accumulating extensive experience in TEM and TEO (transanal endoscopic operations), we have designed a new technical variant of hybrid NOTES: Ta-TME using TEO equipment [14]. The technical benefits of Ta-TME have been described and acknowledged, but several issues remain unresolved. One of the questions to be determined is whether this new technique obtains the same oncological results as conventional techniques. The effect of transanal surgery on sphincter function and its impact on quality of life also need to be established [15].

To answer these questions, we have designed a prospective, randomized multicenter study in collaboration with other centers in Catalonia to compare laparoscopic low anterior resection (L-LAR) with Ta-TME. This study is currently ongoing. The first patient was included in April 2015 and we are still recruiting patients. We expect to finish at the end of 2019.

Methods/design

This is a multicenter, prospective, randomized, controlled study. Candidate participants will be randomized and assigned 1:1 to one of the two arms of the study: the study arm (Ta-TME) and the control arm (L-LAR).

Hypothesis and objectives

The hypothesis is that transanal total mesorectal excision (Ta-TME) achieves faster patient recovery than laparoscopic lower anterior resection (L-LAR) in rectal cancer since the incidence of conversion to open surgery is significantly reduced, and the same anatomopathological and functional results and quality of life are maintained.

The main objective is to demonstrate that Ta-TME reduces the incidence of conversion to open surgery and thereby improves patient recovery. By decreasing the rate of conversion to open surgery, we will achieve a faster recovery. All the patients are enrolled on a fast track program run in conjunction by the four hospitals. This program achieves earlier oral tolerance, good intestinal function, deambulation, and good pain control. All these features together permit earlier patient discharge.

The secondary objective is to assess whether Ta-TME obtains the same anatomopathological, functional and quality of life, and oncological results as L-LAR.

Study setting and participants

Patients are recruited at four colorectal surgery units of acute-care secondary and tertiary hospitals in Catalonia, Spain. All institutions belong to the Spanish public health system. All patients are assessed by the multidisciplinary committee of colorectal tumors of their hospital. The participating hospitals are Parc Taulí University Hospital, the General Hospital of Catalonia, Consorci Sanitari Terrassa, and Mutua Terrassa University Hospital.

Inclusion criteria Inclusion criteria include age over 18; rectal cancer stage cT1-2-3, cN0-1, cM0; lesions less than 10 cm from the anal verge which are candidates for TME and anastomosis, with or without preoperative chemotherapy-radiotherapy; medium- or low-grade adenocarcinoma; and ASA I, II, and III.

Exclusion criteria Exclusion criteria include failure to sign informed consent, pregnancy, hepatic cirrhosis, undifferentiated adenocarcinoma, cT4 stage, metastatic disease (M1), chronic renal failure under treatment with dialysis, ASA IV, and BMI < 18 or > 35 kg/m^2.

Withdrawal criteria Withdrawal criteria include logistical problems performing the randomized technique, unwillingness to continue the trial, intraoperative observation of one of the exclusion criteria, and violation of the study protocol.

Ethics, informed consent, and legal considerations

Patients who meet the inclusion criteria and sign the informed consent enter the randomization process. Patients are informed of the nature, duration, and purpose of the study and, in addition, of all the possible inconveniences that might be reasonably expected. They also receive information in writing. Participants must have the legal capacity to give their consent and exercise their freedom of decision. They have the right to withdraw from the trial at any time.

FOR APPROVAL

The processing of the data compiled by the main data manager during the trial will comply with the current legislation regarding data protection. Patients are identified in the registers by their code number only. Their anonymity is guaranteed. All communication is between the patient and the researcher; there will be no communication with the study sponsor.

The study protocol, the information for patients, and the informed consent documents have been approved by the Clinical Research Ethics Committees of all the participating trial centers, in accordance with Royal Decree 1090/2015, of December 4. The Ethics Committee of the Parc Taulí University Hospital is the committee of reference (ID: 2014/064). The trial has been registered in the ClinicalTrials.gov database (ID: NCT02550769).

The trial is carried out in accordance with the 7th revision of the Declaration of Helsinki [16] and the 2013 SPIRIT Standard Protocol Articles for Clinical Trials. [17].

Description of randomization

Patients are randomly assigned to one or the other group (1:1) using a computer-generated list of random numbers. Randomization is centralized by the main data manager, who determines whether the patient is assigned to the L-LAR control group or the Ta-TME study group.

Trial interventions

Patients diagnosed with rectal adenocarcinoma using full colonoscopy, biopsy, and the rest of the relevant examinations (chest and abdominal CT, rectal MRI, endorectal ultrasound), who are candidates for low anterior resection with or without preoperative chemoradiotherapy and protective ileostomy, are eligible for the study.

After signing the informed consent documentation, patients are randomized to one of the two arms of the study and their preoperative data are recorded.

Prior to surgery, all patients undergo mechanical colon preparation. In accordance with the protocol of each center, antibiotic prophylaxis is administered during anesthesia induction, and also thromboembolic prophylaxis.

Ten surgeons from the four different centers are participating, all of them with many years of experience in rectal cancer laparoscopic surgery. At all the centers, there are at least two senior surgeons with a great deal of experience in both laparoscopic and Ta-ETM. So, we always apply the two team approach as described in our previous paper [14].

Surgical technique

For the control group (L-LAR), TME by laparoscopic low anterior resection of the rectum is performed according to the classic standards described by the COLOR II study 2 and Heald 1.

The patient is placed in the Lloyd-Davis position. The pneumoperitoneum is created at 12 mmHg, and a dissection of the splenic flexure of the colon is performed followed by TME with laparoscopic assistance. The rectum is sectioned distally with the endostapler. The specimen is withdrawn through a Pfannenstiel incision, the colon is divided proximally, and the anvil head of the circular stapler is inserted. The intracorporeal anastomosis is created, and the leak tightness is checked. Finally, a protective ileostomy is created in the right iliac fossa.

For the Ta-TME study group, hybrid NOTES Ta-TME, as described in Serra Aracil X et al. [13], was performed.

With the patient in the same position, surgery starts with the abdominal laparoscopy stage at pressures of 12 mmHg. The splenic flexure is dissected and the inferior mesenteric vessels sectioned at their origin, and the conventional pelvic dissection for the TME is started. Simultaneously, via the transanal route, the TEO rectoscope is introduced. To standardize the procedure, we are only using the normal right TEO rectoscope (7.5 or 15 cm) which we normally use for transanal excisions. The pneumorectum is created using a conventional insufflation technique with a pressure of 14 mmHg and the smoke evacuation system incorporated in the TEO (Karl Storz) equipment. After making the purse-string, transanal dissection of the rectum is started 1 cm above the purse-string. The pelvic dissection is continued upwards following the planes of the TME, until the laparoscopic and transanal fields meet. We resect the rectum proximally and the specimen is extracted transanally, protected by a bag. When the specimen extraction cannot be extracted through the anus, a Pfannenstiel mini-laparotomy is planned, and this information is recorded. The anvil head of the main endostapler is introduced through the TEO rectoscope and placed at the proximal end of the colon according to the technique of Akamatsu [18]. A new purse-string is made in the rectal stump and the intracorporeal end to side anastomosis is created using the circular stapler. The integrity of the anastomosis is checked visually using TEO. Finally, the protection ileostomy is created in the right iliac fossa.

We define conversion to open surgery as the need to perform a midline laparotomy or to extend the Pfannenstiel laparotomy by 5 cm.

The patients who are converted to perineal procedure, these are excluded from the protocol analysis but not from the intention to treat analysis.

These cases are not excluded from the trial. What we do is to consider the change and the reason because we cannot proceed with the planned intervention. And if the patient is converted to perineal surgery, this is also recorded.

Follow-up (Fig. 1, flow chart)

During hospitalization, the surgical variables will be recorded. At the first postoperative visit after 30 days, the pathological

FOR APPROVAL

and morbidity and mortality variables will be recorded. Six months after the ileostomy is closed, the quality of life questionnaires QLQ-CR29, QLQ-C30 [19, 20] will be administered and the Low anterior resection syndrome (LARS) score will be used to assess sphincter function [15, 21].

Study endpoints

Principal endpoint

The principal endpoint is the conversion to open surgery.

Secondary endpoints

Secondary endpoints include general morbidity (according to the Clavien-Dindo classification [22] and the Comprehensive Complication Index, CCI [23]), quality of life, and low anterior resection syndrome (quality of life questionnaires QLQ-CR29, QLQ-C30, and LARS score) before treatment and at 6 months after the closure of the protective ileostomy.

Other variables

Demographics include name and surnames, clinical history number, hospital, age (years), sex, ASA, body mass index (BMI), distance from the tumor to the anal verge (cm), neoadjuvant chemotherapy, neoadjuvant radiotherapy, and preoperative clinical stage.

Surgical variables include surgical time (min), form of anastomosis (manual-mechanical), anastomosis type (end-to-end, side-to-end, reservoir), protection ostomy (ileostomy/colostomy), blood loss (ml), need for blood transfusion, dissection of the splenic flexure, and intraoperative complications.

Postoperative and 30-day morbidity and mortality include oral tolerance (1 l/day, PO day), gas expulsion (PO day), bowel movement-ostomy function (PO day), maximum postoperative pain (VAS 1–10), surgical complications, non-surgical complications, reoperation, mortality (30 days), pathology data, oncological follow-up [24], and survival.

Interruption of study

In the case of each patient, completion or non-completion of the study will be recorded.

Sample size

The sample size has been calculated taking the risk of conversion to open surgery as the main variable. Conversion rates of 20% in the L-LAR group and 5% in the Ta-TME group are estimated, and an α risk of 0.05 and 1-β of 0.9. The estimated number of cases to be included is 53 patients per group; with an estimated loss of 10%, the final number required is 116 patients.

Monitoring

Monitoring is centralized by the promoter center. The variables will be recorded in an access database which will be overseen by the data manager at each hospital.

Statistical analysis

All randomized patients will undergo intention to treat analysis. Per protocol analysis will be carried out, in the L-LAR group including all patients who undergo surgery starting with laparoscopy, and in the Ta-TME group all those who undergo surgery starting with the transanal excision. A flow chart will present all the patients included, lost, randomized, and withdrawn, following the CONSORT criteria for clinical trials.

The description of the variables and the statistical analysis will be carried out using the SPSS program version 21. The description of the quantitative variables will be given as means and standard deviations when the distribution is considered normal, and otherwise as medians, and interquartile ranges. The categorical variables will be described in absolute numbers and percentages.

The statistical analysis of the quantitative variables, with independent groups, will be carried out with the Student's *T* test parametric test provided that its conditions for application are met, and otherwise, the non-parametric Mann-Whitney *U* test. For categorical variables, the Pearson X^2 test will be used. The survival analysis of local and systemic recurrence will be carried out using the Kaplan-Meier estimation method and the log-rank test. The results of the statistical tests will be given for a "*p*" value less than 0.05 along with the 95% confidence interval. The analysis of quality of life will be stratified according to groups of patients with morbidity after neoadjuvant and adjuvant therapy.

Availability of data and material Not applicable.

FOR APPROVAL

Discussion

The introduction of NOTES in rectal surgery (the Ta-TME group) represents an advance on laparoscopic rectal surgery (the L-LAR group). NOTES reduces the aggression on the abdominal wall and facilitates distal access to the tumor via the transanal route. With this trial, we aim to demonstrate that this procedure reduces the need for conversion to open surgery; overall morbidity is lower and patient recovery faster.

As several studies have demonstrated, TME achieves good long-term oncological and survival results in patients with rectal cancer [1]. The evidence provided by various authors

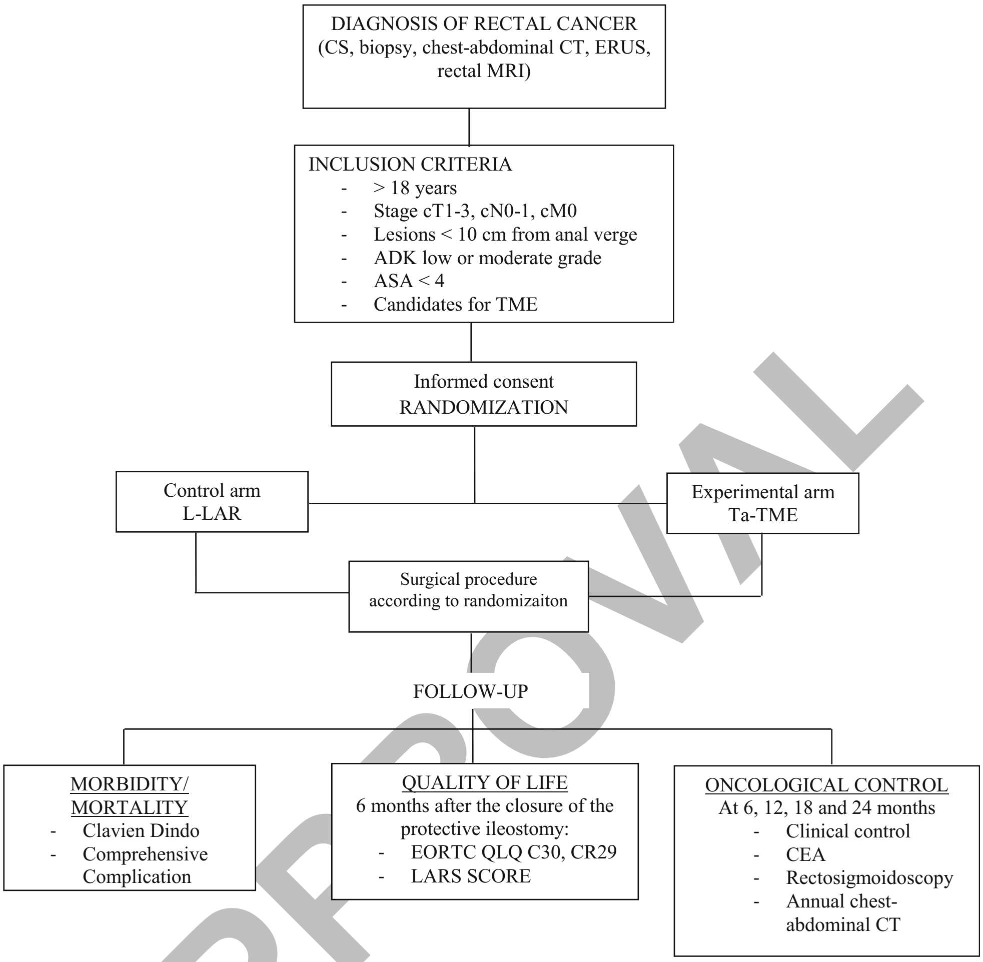

Fig. 1 Flow chart for the diagnosis of rectal cancer. CS, colonoscopy; ERUS, endorectal ultrasound; MRI, magnetic resonance imaging; CT, computerized tomography; ADK, adenocarcinoma. ASA, American Society of Anesthesiologists; L-LAR, laparoscopic low anterior resection; Ta-TME, transanal total mesorectal excision; EORTC QLQ C-30/CR29/LARS score, quality of life questionnaires; CEA, carcinoembryonic antigen

FOR APPROVAL

[2, 25] has confirmed the value of the laparoscopic approach to TME, maintaining the good survival results and improving morbidity and mortality.

L-LAR presents a moderate rate of conversion to open surgery, and these results are similar to those of open surgery with regard to genitourinary alterations, sexual dysfunction, and anorectal functionality [26]. Recent studies report rates of genitourinary alterations and sexual dysfunction of between 20 and 30% in these patients [27, 28], and rates of altered anorectal functionality of between 10 and 30% [29]. All these alterations may negatively affect quality of life. In this study, we aim to analyze a new alternative to TME that might improve quality of life, and to assess the functional results obtained. Even though the quality of life and functional outcomes are not the main objective of the study and the sample size has not been calculated with this aim in mind, the study is likely to provide good descriptive results.

Similarly, although the study is not designed to demonstrate the oncological advantages of the technique, a 3–5-year follow-up study might provide useful insights in this regard.

In patients with colorectal cancer undergoing chemotherapy, the EORTC QLQ-C30 (version 2) quality of life scale gives reliable measurements [30]. For its part, the QLQ-CR29 specifically measures the secondary alterations of colorectal cancer surgery with respect to genitourinary alterations and the effect of the ostomy. Sphincter function after LAR will be measured in both groups with the low anterior resection syndrome score (the LARS questionnaire) [20].

Little information on the effect of Ta-TME on quality of life and anorectal function is currently available. Based on manometric data and quality of life data obtained from quality of life questionnaires, some groups [31, 32] have presented encouraging results and conclude that the quality of life in Ta-TME patients does not differ from that of patients undergoing conventional TME. Indeed, some of the manometric data published indicate that there are few or no variations in anorectal functionality, and that any variations that occur are transient [29].

Conclusions

The introduction of minimally invasive surgical techniques has been one of the major breakthroughs in surgery in recent years. These approaches reduce morbidity, accelerate

recovery, and improve quality of life. In this study, we intend to demonstrate that the introduction of the transanal route in rectal surgery (the Ta-TME group) represents an advance on laparoscopic rectal surgery (the L-LAR group); it provides access to the pelvis from another angle, lessens the aggression to the abdominal wall, and reduces the conversion rate to open surgery. This speeds up patient recovery and decreases overall morbidity, while at the same time maintaining comparable functional results.

Several groups have described the technical advantages of the transanal approach in comparative cohort studies [33]. But the study proposed here will be the first prospective, randomized clinical study to compare Ta-TME with conventional L-LAR, focusing on technical aspects, postoperative morbidity, pathological data, functionality, and quality of life.

Acknowledgements We thank all the members of the multidisciplinary committee for colorectal tumors of the hospital for their support. We thank Michael Maudsley for help with the English translation.

Authors' contributions All authors contributed to the design and writing of the paper. All authors have read and approved the final manuscript.

Funding This study was funded by a grant from the Fundación Parc Taulí Research prize IDCSALUD - Hospital General de Catalunya, 2015.

Compliance with ethical standards

Competing interests The authors declare that they have no competing interests.

Consent for publication Not applicable.

Ethics approval and consent to participate The study has been approved by the local ethics committees of the participating centers.

References

1. Heald RJ, Husband EM, Ryall RD (1982) The mesorectum in rectal cancer surgery—the clue to pelvic recurrence? Br J Surg 69:613–616
2. Jayne D, Pigazzi A, Marshall H et al (2017) Effect of robotic-assisted vs conventional laparoscopic surgery on risk of conversion to open laparotomy among patients undergoing resection for rectal cancer. The ROLARR randomized clinical trial. JAMA 318(16):1569–1580. https://doi.org/10.1001/jama.2017.7219
3. Van der Pas MH, Haglind E, Cuesta MA, Fürst A, Lacy AM, Hop WC, Bonjer HJ, Colorectal cancer Laparoscopic or Open Resection II (COLOR II) Study Group (2013) Laparoscopic versus open surgery for rectal cancer (COLOR II): short-term outcomes of a randomised, phase 3 trial. Lancet Oncol 14:210–218. https://doi.org/10.1016/S1470-2045(13)70016-0
4. Buess G, Hutterer F, Theiss J, Bobel M, Isselhard W, Pichlmaier H (1984) A system for a transanal endoscopic rectum operation. Chirurg 55:677–680
5. Rattner D, Kalloo A; ASGE/SAGES Working Group (2006). ASGE/SAGES Working Group on Natural Orifice Translumenal Endoscopic Surgery. October 2005 Surg Endosc 20: 329-333. https://doi.org/10.1007/s00464-005-3006-0
6. Lehmann KS, Ritz JP, Wibmer A, Gellert K, Zornig C, Burghardt J, Büsing M, Runkel N, Kohlhaw K, Albrecht R, Kirchner TG, Arlt G, Mall JW, Butters M, Bulian DR, Bretschneider J, Holmer C, Buhr HJ (2010) The German registry for natural orifice translumenal endoscopic surgery: report of the first 551 patients. Ann Surg 252:263–270. https://doi.org/10.1097/SLA.0b013e3181e6240f
7. Kalloo AN, Singh VK, Jagannath SB, Niiyama H, Hill SL, Vaughn CA, Magee CA, Kantsevoy SV (2004) Flexible transgastric peritoneoscopy: a novel approach to diagnostic and therapeutic interventions in the peritoneal cavity. Gastrointest Endosc 60:114–117
8. Leroy J, Barry BD, Melani A, Mutter D, Marescaux J (2013) No-scar transanal total mesorectal excision: the last step to pure NOTES for colorectal surgery. JAMA Surg 148:226–230
9. Tuech JJ, Bridoux V, Kianifard B, Schwarz L, Tsilividis B, Huet E, Michot F (2011) Natural orifice total mesorectal excision using transanal port and laparoscopic assistance. Eur J Surg Oncol 37:334–335. https://doi.org/10.1016/j.ejso.2010.12.016
10. Zhang H, Zhang YS, Jin XW, Li MZ, Fan JS, Yang ZH (2013) Transanal single-port laparoscopic total mesorectal excision in the treatment of rectal cancer. Tech Coloproctol 17:117–123. https://doi.org/10.1007/s10151-012-0882-x
11. Pearl JP, Marks JM, Ponsky JL (2008) Hybrid surgery: combined laparoscopy and natural orifice surgery. Gastrointest Endosc Clin N Am 18:325–332. https://doi.org/10.1016/j.giec.2008.01.006
12. Horgan S, Cullen JP, Talamini MA, Mintz Y, Ferreres A, Jacobsen GR, Sandler B, Bosia J, Savides T, Easter DW, Savu MK, Ramamoorthy SL, Whitcomb E, Agarwal S, Lukacz E, Dominguez G, Ferraina P (2009) Natural orifice surgery: initial clinical experience. SurgEndosc 23:1512–1518. https://doi.org/10.1007/s00464-009-0428-0
13. Sylla P, Rattner DW, Delgado S, Lacy AM (2010) NOTES transanal rectal cancer resection using transanal endoscopic microsurgery and laparoscopic assistance. Surg Endosc 24:1205–1210. https://doi.org/10.1007/s00464-010-0965-6
14. Serra-Aracil X, Mora-López L, Casalots A, Pericay C, Guerrero R, Navarro-Soto S Hybrid-NOTES: TEO for transanal total mesorectal excision. Intracorporeal resection and anastomosis. Surg Endosc 30(1):346–354. https://doi.org/10.1007/s00464-015-4170-5
15. Emmertsen KJ, Laurberg S (2012) Low anterior resection syndrome score: development and validation of a symptom-based scoring system for bowel dysfunction after low anterior resection for rectal cancer. Ann Surg 255:922–928. https://doi.org/10.1097/SLA.0b013e31824f1c21
16. Emanuel EJ (2013) Reconsidering the Declaration of Helsinki. Lancet 381:1532–1533
17. Chan A-W, Tetzlaff JM, Altman DG, Laupacis A, Gøtzsche PC, Krleža-Jerić K, Hróbjartsson A, Mann H, Dickersin K, Berlin JA, Doré CJ, Parulekar WR, Summerskill WSM, Groves T, Schulz KF, Sox HC, Rockhold FW, Rennie D, Moher D (2013) SPIRIT 2013 statement: defining standard protocol items for clinical trials. Ann Intern Med 158:200–207. https://doi.org/10.7326/0003-4819-158-3-201302050-00583
18. Akamatsu H, Omori T, Oyama T, Tori M, Ueshima S, Nishida T, Nakahara M, Abe T (2009) Totally laparoscopic low anterior resection for lower rectal cancer: combination of a new technique for intracorporeal anastomosis with prolapsing technique. Dig Surg 26:446–450. https://doi.org/10.1159/000239761
19. Arraras JI, Suárez J, Arias de la Vega F, Vera R, Asín G, Arrazubi V, Rico M, Teijeira L, Azparren J (2011) The EORTC Quality of Life questionnaire for patients with colorectal cancer: EORTC QLQ-CR29 validation study for Spanish patients. Clin Transl Oncol 13(1):50–56. https://doi.org/10.1007/s12094-011-0616-y
20. Gujral S, Conroy T, Fleissner C, Sezer O, King PM, Avery KN, Sylvester P, Koller M, Sprangers MA, Blazeby JM (2007)

FOR APPROVAL

Assessing quality of life in patients with colorectal cancer: an update of the EORTC quality of life questionnaire. European Organisation for Research and Treatment of Cancer Quality of Life Group. Eur J Cancer 43(10):1564–1573. https://doi.org/10.1016/j.ejca.2007.04.005

21. Juul T, Ahlberg M, Biondo S, Emmertsen KJ, Espin E, Jimenez LM, Matzel KE, Palmer G, Sauermann A, Trenti L, Zhang W, Laurberg S, Christensen P (2014) International validation of the low anterior resection syndrome score. Ann Surg 259(4):728–734. https://doi.org/10.1097/SLA.0b013e31828fac0b
22. Dindo D, Demartines N, Clavien P-A (2004) Classification of surgical complications. A new proposal with evaluation in a cohort of 6336 patients and results of a survey. Ann Surg 240:205–213
23. Slankamenac K, Graf R, Barkun J et al (2013) The comprehensive complication index: a novel continuous scale to measure surgical morbidity. Ann Surg 258:1–7. https://doi.org/10.1097/SLA.0b013e318283669c
24. Quirke P, Dixon MF, Durdey P, Williams NS (1986) Local recurrence of rectal adenocarcinoma due to inadequate surgical resection. Histopathological study of lateral tumour spread and surgical excision. Lancet 1;2(8514):996–999
25. Enker WE, Merchant N, Cohen AM (1999) Safety and efficacy of low anterior resection for rectal cancer: 681 consecutive cases from a specialty service. Ann Surg 230(4):544e552
26. Andersson J, Abis G, Gellerstedt M, Angenete E, Angerås U, Cuesta M A, Jess P, Rosenberg J, Bonjer H. J, Haglind E. (2014) Patient-reported genitourinary dysfunction after laparoscopic and open rectal cancer surgery in a randomized trial (COLOR II). Br J Surg, 101: 1272–1279. https://doi.org/10.1002/bjs.9550
27. Kneist W, Junginger T (2004) Residual urine volume after total mesorectal excision: an indicator of pelvic autonomic nerve preservation? Results of a case-control study. Colorectal Dis6:432–437. https://doi.org/10.1111/j.1463-1318.2004.00711.x
28. Shah EF, Huddy SPJ (2001) A prospective study of genito-urinary dysfunction after surgery for colorectal cancer. Color Dis 3:122–125
29. Aaronson NK, Ahmedzai S, Bergman B, Bullinger M, Cull A, Duez NJ, Filiberti A, Flechtner H, Fleishman SB, de Haes JC, et al. (1993) The European Organization for Research and Treatment of Cancer QLQ-C30: a quality-of-life instrument for use in international clinical trials in oncology J Natl Cancer Inst 3;85(5):365–376
30. Steinemann DC, Zerz A, Germann S, Lamm SH (2016) Anorectal function and quality of life after transrectal rigid-hybrid natural orifice translumenal endoscopic sigmoidectomy. J Am Coll Surg 223(2):299–307. https://doi.org/10.1016/j.jamcollsurg.2016.04.002
31. Koedam TW, van Ramshorst GH, Deijen CL, Elfrink AK, Meijerink WJ, Bonjer HJ, Sietses C, Tuynman JB (2017) Transanal total mesorectalexcision (TaTME) for rectal cancer: effects on patient-reported quality of life and functional outcome. Tech Coloproctol 21(1):25–33. https://doi.org/10.1007/s10151-016-1570-z
32. Fernández-Hevia M, Delgado S, Castells A, Tasende M, Momblan D, Díaz del Gobbo G, DeLacy B, Balust J, Lacy AM (2015) Transanal total mesorectal excision in rectal cancer: short-term outcomes in comparison with laparoscopic surgery. Ann Surg 261(2):221–227. https://doi.org/10.1097/SLA.0000000000000865

FOR APPROVAL
