# Supplementary material for: Quality of life, bowel dysfunction, and long-term oncological outcomes after transanal versus laparoscopic total mesorectal excision for mid- and low- rectal cancer (Ta-LaTME study): multicentre randomized open-label trial
Source: BJS Open. 2026 Aug 4;10(4):zrag045. doi: 10.1093/bjsopen/zrag045 (PMC13436816; doi:10.1093/bjsopen/zrag045)
Supplement: Supplementary file 1 [file zrag045_supplementary_data.zip › Supplementary_Material.docx]

**Title**

**Quality of life, bowel dysfunction, and long-term oncological outcomes after transanal versus laparoscopic total mesorectal excision for mid and low rectal cancer (Ta-LaTME study): Multicentre randomized open-label trial.**

Authors

Xavier Serra-Aracil, PhD1; Anna Gonzalez MD2; Josep Bargalló, MD2; Anna Serracant, PhD 1; Jordi Roura, MD3; Ladislao Cayetano-Paniagua, MD2; Salvadora Delgado, PhD4; Alba Zarate, PhD 5 for the Ta-LaTME study Group

1Coloproctology Unit. Parc Tauli University Hospital, Sabadell. Institut d'investigació i innovació Parc Tauli I3PT. Department of Surgery, Universitat Autònoma de Barcelona. 2 General and Digestive Surgery Department, Hospital Consorcio Sanitario Terrassa, Terrassa, Spain. 3 General and Digestive Surgery Department, Hospital General de Catalunya, Barcelona, Spain. 4 Coloproctology Unit, General and Digestive Surgery Department, Hospital Universitario Mutua de Terrassa, Terrassa, Spain. 5 General and Digestive Surgery Department, Germans Trias i Pujol University Hospital, Badalona, Spain.

**Corresponding author.**

Xavier Serra-Aracil

Email: javier.serra@uab.cat

Coloproctology Unit. General and Digestive Surgery Service.

Parc Tauli University Hospital, Sabadell. Institut d'investigació i innovació Parc Tauli I3PT

Professor Department of Surgery, Universitat Autònoma de Barcelona.

Parc Tauli s/n, 08208 Sabadell (Barcelona). Spain

Phone number: +34 937 23 10 10. Ext 21490

ORCID ID: IDorcid.org/0000-0003-0291-1900

**Supplementary Materials - Index**

**Supplementary Tables**

| Table S1. Patients’ characteristics with valid and no valid questionnaires EORTC QLQ-C30/CR29 | Pag. 3 |
| --- | --- |
| Table S2. Preoperative and postoperative EORTC QLQ-C30 results (PP population) | Pag. 4 |
| Table S3 Preoperative and postoperative EORTC QLQ-CR29 results (PP population) | Pag. 5 |
| Table S4. Patients’ characteristics with valid and no valid questionnaires LARS | Pag. 6 |
| Table S5. Preop/Postop: LARS score, mITT | Pag. 7 |
| Table S6. Preop/Postop LARS score, PP population | Pag. 8 |
| Table S7. Univariate analysis of Major LARS (Modified Intention-to-Treat) | Pag. 9 |

**Supplementary Figures**

| **Figure legend S1.** Flow chart for the diagnosis of rectal cancer. CS, colonoscopy; ERUS, endorectal ultrasound; MRI, magnetic resonance imaging; CT, computed tomography. ADK, adenocarcinoma. ASA, American Society of Anaesthesiologists; LLAR, laparoscopic low anterior resection; TaTME, transanal total mesorectal excision; EORTC QLQ C-30/CR29/LARS score, quality of life questionnaires; CEA, carcinoembryonic antigen | Pag. 10 |
| --- | --- |
| **Figure legend S2**. CONSORT 2010 Flow Diagram Ta-LaTME study | Pag. 11 |
| **Figure legend S3.** A1: QLQ-C30-Global Health-Functional Scales, LaTME. mITT (Median). A2: QLQ- C30-Symptoms, LaTME. mITT (Median). B1: QLQ-C30-Global Health-Functional Scales, TaTME. mITT (Median). B2: QLQ- C30-Symptoms. TaTME. mITT (Median) | Pag. 12 |
| **Figure legend S4.** A1: QLQ-CR29-Functional Scales. TaTME. mITT (Median). A2: QLQ- CR29-Symptoms. LaTME. mITT (Median). B1: QLQ-CR29-Functional Scales. LaTME. mITT (Median). B2: QLQ- CR29-Symptoms. TaTME. mITT (Median. | Pag. 13 |
| **Figure legend S5.** A1: LARS LaTME. mITT (Median). A2: LARS TaTME. mITT (Median). B: LARS. Preop_. LaTME vs TaTME. mITT (Median) | Pag. 14 |

**Table S1. Patients’ characteristics with valid and no valid questionnaires EORTC QLQ-C30/CR29**

| **Baseline characteristics**  **All patients (n=105)** | | | **Valid questionnaires (n=67, 63.8%)** | **No valid questionnaires (n=38, 38.2%)** | **P value** |
| --- | --- | --- | --- | --- | --- |
| Sex | | Women | 20 (29.9%) | 10 (26.3) | 0.82^a^ |
|  |  | Men | 47 (70.1) | 28 (73.1) |  |
| Age, median (IQR) years | | | 65 (17) | 72.5 (18) | 0.01^b^ |
| BMI, median (IQR) | | | 27 (5.9) | 25.6 (5.5) | 0.13 ^b^ |
| ASA score | | I | 3 (4.5) | 2 (5.3) | <0.01 |
|  |  | II | 47 (70.1) | 14 (36.8) |  |
|  |  | III | 17 (24.4) | 22 (57.9) |  |
| Neoadjuvant treatment (n=65) | | | 17 (44.7) | 48 (71.6) | 0.01 ^a^ |
| Clinical TNM | | T1 | 1 (1.5) | 2 (5.3) | 0.49 ^a^ |
| T  N | | T2 | 10 (14.9) | 8 (21.1) |  |
|  |  | T3ab | 30 (44.8) | 17 (44.7) |  |
|  |  | T3cd | 26 (38.8) | 11 (28.9) |  |
|  |  | N0 | 29 (43.3) | 22 (57.9) | 0.33 ^a^ |
|  |  | N1 | 28 (41.8) | 11 (28.9) |  |
|  |  | N2 | 10 (14.9) | 5 (13.2) |  |
| Tumour height, median (IQR), cm | | | 9 (3) | 8 (3) | 0.58 ^b^ |
| LaTME/TaTME | LaTME | | 34 (50.7) | 16 (42.1) | 0.42 ^a^ |
|  | TaTME | | 33 (49.3) | 22 (57.9) |  |
| Defunctioning stoma | Ileostomy | | 64 (95.5) | 33 (86.8) | 0.21 ^a^ |
|  | Colostomy | | 1 (1.5) | 3 (7.9) |  |
|  | None | | 2 (3) | 2 (5.3) |  |
| Overall morbidity | | | 21 (31.3) | 18 (47.3) | 0.09 ^a^ |
| Relevant morbidity (Cl–D > II) | | | 3 (4.5) | 7 (18.4) | 0.03 ^a^ |
| Anastomotic leak | | | 1 (2.6) | 6 (9) | 0.21 ^a^ |
| Time to stoma closure median (IQR) (months) | | | 13 (1) | 13 (0.5) | 0.22 ^b^ |
| Follow-up time, median (IQR) (months) | | | 61.5 (28) | 43 (33) | <0.01 ^b^ |
| Local recurrence (n=101) | | | 1 (1.5) | 4 (11.8) | 0.04 ^a^ |
| Distant recurrence (n=101) | | | 5 (7.5) | 12 (35.3) | 0.01 ^a^ |
| Disease-free survival (n=101) | | | 58 (86.6) | 8 (23.5) | < 0.01 ^a^ |
| Overall survival (n=101) | | | 64 (95.5) | 9 (26.5) | < 0.01 ^a^ |

LaTME: Laparoscopic Total Mesorectal Excision. TaTME: Transanal Total Mesorectal Excision. IQR: Interquartile Range. BMI: Body Mass Index. ^a^ Pearson chi-square or Fisher’s exact test. ^b^ Mann-Whitney U Test

**Table S2. Preoperative and postoperative EORTC QLQ-C30 results (PP population)**

| **Characteristics** | | **LaTME**  **(n=40)**  **Median (IQR)** | **CID- LaTME** | **ES- LaTME** | **SRM- LaTME** | **P value**  **Pre/Post**  **LaTME** | **TaTME**  **(n=54)**  **Median (IQR)** | **CID- TaTME** | **ES- TaTME** | **SRM- TaTME** | **P value**  **Pre/Post**  **TaTME** | **P value**  **Pre/Post**  **LaTME / TaTME** |
| --- | --- | --- | --- | --- | --- | --- | --- | --- | --- | --- | --- | --- |
| **Global health status** | Preop. | 75 (41.7) | -4.30 | -0.19 | -0.18 | 0.489^ | 83.3 (33.3) | 7.83 | 0.45 | 0.29 | 0.108^ | 0.336* |
|  | Postop. | 83.3 (25) |  |  |  |  | 83.3 (16.7) |  |  |  |  | 0.236* |
| **Functional Scales** | | | | | | | | | | | | |
| Physical functioning | Preop. | 100 (13.3) | -3.66 | -0.28 | -0.25 | 0.194^ | 100 (3.3) | 1.21 | 0.07 | 0.07 | 0.314^ | 0.172* |
|  | Postop | 100 (6.7) |  |  |  |  | 100 (13.3) |  |  |  |  | 0.117* |
| Role functioning | Preop. | 100 (0) | 3.23 | 0.22 | 0.12 | 0.526^ | 100 (0) | 2.02 | 0.13 | 0.07 | 0.812^ | 0.629* |
|  | Postop | 100 (0) |  |  |  |  | 100 (0) |  |  |  |  | 0.700* |
| Emotional functioning | Preop. | 83.3 (50) | -15.59 | -0.56 | -0.49 | 0.017^ | 83.3 (8.3) | -8.59 | -0.49 | -0.32 | 0.052^ | 0.978* |
|  | Postop. | 100 (16.7) |  |  |  |  | 100 (16.7) |  |  |  |  | 0.393* |
| Cognitive functioning | Preop. | 100 (16.7) | -6.45 | -0.33 | -0.36 | 0.055^ | 100 (8.3) | 5.05 | 0.38 | 0.26 | 0.157^ | 0.440* |
|  | Postop. | 100 (0) |  |  |  |  | 100 (25) |  |  |  |  | 0.017* |
| Social functioning | Preop. | 100 (16.67) | 5.91 | 0.26 | 0.19 | 0.506^ | 100 (16.7) | 1.01 | 0.04 | 0.03 | 0.981^ | 0.665* |
|  | Postop. | 100 (33.3) |  |  |  |  | 100 (33.3) |  |  |  |  | 0.704* |
| **Symptom Scales** | | | | | | | | | | | | |
| Fatigue | Preop. | 22.2 (33.3) | 8.96 | 0.40 | 0.35 | 0.042^ | 0 (27.8) | -3.70 | -0.18 | -0.12 | 0.438^ | 0.070* |
|  | Postop. | 0 (22.2) |  |  |  |  | 11.1 (27.8) |  |  |  |  | 0.349* |
| Nausea and vomiting | Preop. | 0 (0) | 2.69 | 0.22 | 0.25 | 0.180^ | 0 (0) | -1.01 | -0.21 | -0.09 | 0.680^ | 0.762* |
|  | Postop. | 0 (0) |  |  |  |  | 0 (0) |  |  |  |  | 0.570* |
| Pain | Preop. | 0 (33.3) | 8.60 | 0.7 | 0.25 | 0.124^ | 0 (8.3) | -5.05 | -0.38 | -0.24 | 0.217^ | 0.031* |
|  | Postop. | 0 (0) |  |  |  |  | 0 (16.7) |  |  |  |  | 0.500* |
| Dyspnoea | Preop. | 0 (0) | 2.98 | 0.15 | 0.12 | 0.534^ | 0 (0) | -1.01 | -0.10 | -0.05 | 1.0^ | 0.118* |
|  | Postop. | 0 (0) |  |  |  |  | 0 (0) |  |  |  |  | 0.198* |
| Insomnia | Preop. | 0 (33.3) | 6.45 | 0.20 | 0.21 | 0.194^ | 0 (33.3) | 8.08 | 0.24 | 0.19 | 0.374^ | 0.907* |
|  | Postop. | 0 (33.3) |  |  |  |  | 0 (0) |  |  |  |  | 0.461* |
| Appetite loss | Preop. | 0 (33.3) | 6.45 | 0.32 | 0.30 | 0.196^ | 0 (0) | -5.05 | -0.3 | -0.2 | 0.473^ | 0.099* |
|  | Postop. | 0 (0) |  |  |  |  | 0 (16.7) |  |  |  |  | 0.137* |
| Constipation | Preop. | 0 (33.3) | 1.08 | 0.06 | 0.04 | 0.924^ | 0 (0) | -14.14 | -0.53 | -0.32 | 0.077^ | 0.037* |
|  | Postop. | 0 (33.3) |  |  |  |  | 0 (50) |  |  |  |  | 0.792* |
| Diarrhoea | Preop. | 0 (66.7) | 1.08 | 0.03 | 0.03 | 0.853^ | 0 (33.3) | -17.17 | -0.52 | -0.34 | 0.068^ | 0.710* |
|  | Postop. | 0 (33.3) |  |  |  |  | 33.3 (83.3) |  |  |  |  | 0.252* |
| Financial difficulties | Preop. | 0 (33.3) | 4.3 | 0.13 | 0.13 | 0.556^ | 0 (50) | 8.08 | 0.23 | 0.23 | 0.152^ | 0.828* |
|  | Postop. | 0 (0) |  |  |  |  | 0 (16.7) |  |  |  |  | 0.935* |

Data are expressed as median (IQR). ^ Wilcoxon signed-rank test (within-group comparisons). * Mann–Whitney U test (between-group comparisons). CID = clinically important difference; ES = effect size; SRM = standardized response mean.

**Table S3 Preoperative and postoperative EORTC QLQ-CR29 results (PP population)**

| **Characteristics** | | **LaTME**  **(n=40)**  **Median (IQR)** | **CID- LaTME** | **ES- LaTME** | **SRM- LaTME** | **P value**  **Pre/Post**  **LaTME** | **TaTME**  **(n=54)**  **Median (IQR)** | **CID- TaTME** | **ES - TaTME** | **SRM- TaTME** | **P value**  **Pre/Post**  **TaTME** | **P value**  **Pre/Post**  **LaTME/TaTME** |
| --- | --- | --- | --- | --- | --- | --- | --- | --- | --- | --- | --- | --- |
| **Functional Scales** | | | | | | | | | | | | |
| Body Image | Preop. | 100 (22.2) | -3.58 | -0.22 | -0.12 | 0.233^ | 100 (11.1) | 1.16 | -0.13 | -0.13 | 0.548^ | 0.297* |
|  | Postop. | 100 (0) |  |  |  |  | 100 (11.1) |  |  |  |  | 0.486* |
| Sexual function (men) | Preop. | 66.7 (16.7) | -2.07 | 0 | 0 | 0.672^ | 66.7 (33.3) | 0.51 | 0.07 | 0.05 | 0.618^ | 0.305* |
|  | Postop. | 66.7 (66.7) |  |  |  |  | 66.7 (33.3) |  |  |  |  | 0.364* |
| Sexual function (women) | Preop. | 100 (33.3) | 3.91 | 0.17 | 0.2 | 1.0^ | 66.7 (33.3) | -19.05 | -0.83 | -1.07 | 0.059^ | 0.252* |
|  | Postop. | 83.3 (33.3) |  |  |  |  | 100 (33.3) |  |  |  |  | 0.475* |
| **Symptom Scales** | | | | | | | | | | | | |
| Urinary frequency | Preop. | 33.3 (50) | 20.43 | 0.7 | 0.54 | 0.006^ | 0 (50) | 2.02 | 0.07 | 0.05 | 0.786^ | 0.104* |
|  | Postop. | 0 (33.3) |  |  |  |  | 16.7 (33.3) |  |  |  |  | 0.176* |
| Blood and mucus with stool | Preop. | 16.7 (50) | 29.03 | 0.96 | 1.0 | 0.0^ | 33.3 (50) | 23.74 | 0.85 | 0.75 | 0.001^ | 0.792* |
|  | Postop. | 0 (0) |  |  |  |  | 0 (0) |  |  |  |  | 0.053* |
| Stool Frequency | Preop. | 16.7 (33.3) | -5.75 | -0.31 | -0.18 | 0.581^ | 8.3 (16.7) | -21.43 | -0.88 | -0.52 | 0.017^ | 0.484* |
|  | Postop. | 16.7 (33.3) |  |  |  |  | 33.3 (45.8) |  |  |  |  | 0.028* |
| Urinary Incontinence | Preop. | 0 (0) | -1.08 | -0.05 | -0.04 | 0.862^ | 0 (0) | -8.08 | -0.42 | -0.39 | 0.029^ | 0.795* |
|  | Postop. | 0 (0) |  |  |  |  | 0 (33.3) |  |  |  |  | 0.197* |
| Dysuria | Preop. | 0 (0) | 0 | 0 | 0 | 1.0^ | 0 (0) | -5.05 | --- | -0.27 | 0.102^ | 0.141* |
|  | Postop. | 0 (0) |  |  |  |  | 0 (0) |  |  |  |  | 0.675* |
| Abdominal pain | Preop. | 0 (33.3) | 6.45 | 0.24 | 0.2 | 0.294^ | 0 (33.3) | 3.03 | 0.11 | 0.09 | 0.557^ | 0.447* |
|  | Postop. | 0 (33.3) |  |  |  |  | 0 (16.7) |  |  |  |  | 0.936* |
| Buttock pain | Preop. | 0 (33.3) | 1.08 | 0.04 | 0.03 | 0.958^ | 0 (0) | -9.09 | -0.62 | -0.27 | 0.146^ | 0.090* |
|  | Postop. | 0 (33.3) |  |  |  |  | 0.0 (25) |  |  |  |  | 0.896* |
| Bloated feeling | Preop. | 0 (33.3) | 7.53 | 0.3 | 0.25 | 0.185^ | 0 (33.3) | 1.01 | 0.03 | 0.03 | 0.936^ | 0.861* |
|  | Postop. | 0 (0) |  |  |  |  | 0 (33.3) |  |  |  |  | 0.340* |
| Dry mouth | Preop. | 0 (66.7) | 15.05 | 0.44 | 0.41 | 0.038^ | 0 (33.3) | -3.03 | -0.11 | -0.08 | 0.675^ | 0.186* |
|  | Postop. | 0 (33.3) |  |  |  |  | 0 (33.3) |  |  |  |  | 0.423* |
| Hair loss | Preop. | 0 (0) | 2.15 | 0.08 | 0.06 | 0.762^ | 0 (33.3) | 6.06 | 0.24 | 0.25 | 0.403^ | 0.278* |
|  | Postop. | 0 (0) |  |  |  |  | 0 (0) |  |  |  |  | 0.915* |
| Trouble with taste | Preop. | 0 (0) | 8.60 | 0.31 | 0.25 | 0.207^ | 0 (16.7) | 3.03 | 0.09 | 0.08 | 0.776^ | 0.807* |
|  | Postop. | 0 (0) |  |  |  |  | 0 (0) |  |  |  |  | 0.239* |
| Anxiety | Preop. | 66.7 (66.7) | -18.28 | -0.54 | -0.48 | 0.008^ | 66.7 (66.7) | -10.23 | -0.38 | -0.29 | 0.277^ | 0.285* |
|  | Postop. | 66.7 (33.3) |  |  |  |  | 66.7 (33.3) |  |  |  |  | 0.960* |
| Weight | Preop. | 100 (33.3) | -4.3 | -0.17 | -0.14 | 0.417^ | 100 (33.3) | -1.92 | -0.08 | -0.07 | 0.892^ | 0.993* |
|  | Postop. | 100 (33.3) |  |  |  |  | 100 (0) |  |  |  |  | 0.669* |
| Flatulence | Preop. | 33.3 (66.7) | -2.95 | -0.08 | -0.06 | 0.917^ | 0 (33.3) | -27.08 | -0.89 | -0.60 | 0.008^ | 0.132* |
|  | Postop. | 33.3 (33.3) |  |  |  |  | 33.3 (66.7) |  |  |  |  | 0.143* |
| Faecal Incontinence | Preop. | 0 (33.3) | 0.08 | -0.1 | -0.06 | 0.676^ | 0 (0) | -29.91 | -1.03 | -0.77 | 0.002^ | 0.061* |
|  | Postop. | 0 (33.3) |  |  |  |  | 33.3 (66.7) |  |  |  |  | 0.010* |
| Sore Skin | Preop. | 0 (33.3) | -7.17 | -0.48 | -0.27 | 0.188^ | 0 (0) | -28.87 | -2.08 | -0.61 | 0.006^ | 0.024* |
|  | Postop. | 0 (33.3) |  |  |  |  | 0 (66.7) |  |  |  |  | 0.341* |
| Embarrassment by bowel movement | Preop. | 0 (0) | -6.41 | -0.42 | -0.18 | 0.445^ | 0 (0) | -13.1 | -0.69 | -0.53 | 0.013^ | 0.049* |
|  | Postop. | 0 (33.3) |  |  |  |  | 0 (33.3) |  |  |  |  | 0.330* |
| Stoma care problems | Preop. | --- | --- | --- | --- | --- | --- | --- | --- | --- | --- | --- |
|  | Postop. | --- |  |  |  |  | --- |  |  |  |  | --- |
| Impotence | Preop. | 33.3 (66.7) | 7.84 | 0.21 | 0.18 | 0.463^ | 0 (16.7) | -21.95 | -0.81 | -0.67 | 0.011^ | 0.064* |
|  | Postop. | 33.3 (33.3) |  |  |  |  | 33.3 (66.7) |  |  |  |  | 0.588* |
| Dyspareunia | Preop. | 0 (33.3) | -3.81 | -0.68 | -0.41 | 0.317^ | 0 (66.7) | 14.29 | 0.34 | 0.38 | 0.317^ | 0.805* |
|  | Postop. | 0 (33.3) |  |  |  |  | 0 (0) |  |  |  |  | 0.669* |

^Test de Wilcoxon para muestras apareadas. *Test de Mann-Whitney para muestras independientes. CID: Diferencia Clínicamente Importante. ES: Tamaño del Efecto. SRM: Métrica de Respuesta Estandarizada

**Table S4. Patients’ characteristics with valid and no valid questionnaires LARS**

| **Baseline characteristics**  **All patients (n=105)** | | | **Valid questionnaires (n=72, 68.6%)** | **NO valid questionnaires (n=33, 31.4%)** | **P value** |
| --- | --- | --- | --- | --- | --- |
| Sex | | Women | 20 (27.8) | 10 (30.3) | 0.82 ^a^ |
|  |  | Men | 52 (72.2) | 23 (69.7) |  |
| Age, median (IQR) years | | | 66.5 (17) | 72 (15) | 0.07 ^b^ |
| BMI, median (IQR) | | | 26.7 (5.7) | 25.7 (5.4) | 0.59 ^b^ |
| ASA score | | I | 4 (5.6) | 1 (3) | 0.01 ^a^ |
|  |  | II | 48 (66.7) | 13 (39.4) |  |
|  |  | III | 20 (27.8) | 19 (57.6) |  |
| Neoadjuvant treatment (n=65) | | | 51 (70.8) | 14 (42.4) | 0.01 ^a^ |
| Clinical TNM | | T1 | 1 (1.4) | 2(6.1) | 0.43 ^a^ |
| T  N | | T2 | 12 (16.7) | 6 (18.2) |  |
|  |  | T3ab | 31 (43.1) | 16 (48.5) |  |
|  |  | T3cd | 28 (38.9) | 9 (27.3) |  |
|  |  | N0 | 30 (41.7) | 21 (63.6) | 0.1 ^a^ |
|  |  | N1 | 31 (43.1) | 8 (24.2) |  |
|  |  | N2 | 11 (15.3) | 4 (12.1) |  |
| Tumour height, median (IQR), cm | | | 9 (3) | 8 (3) | 0.46 ^b^ |
| LaTME/TaTME | LaTME | | 33 (45.8) | 17 (51.5) | 0.68 ^a^ |
|  | TaTME | | 39 (54.2) | 16 (48.5) |  |
| Defunctioning stoma | Ileostomy | | 68 (94.4) | 29 (87.9) | 0.16 ^a^ |
|  | Colostomy | | 1 (1.4) | 3 (9.1) |  |
|  | None | | 3 (4.2) | 1 (3) |  |
| Overall morbidity | | | 27 (37.5) | 12 (38) | 0.83 ^a^ |
| Relevant morbidity (Cl–D > II) | | | 4 (5.6) | 6 (18.2) | 0.07 ^a^ |
| Anastomotic leak | | | 6 (8.3) | 1 (3) | 0.43 ^a^ |
| Time ostomy closure median (IQR) (months) | | | 13 (1) | 13 (0.5) | 0.33 ^b^ |
| Time follows up (months) | | | 60 (27) | 42(29) | <0.01 ^b^ |
| Local recurrence (n=101) | | | 2 (2.8) | 3 (10.3) | 0.14 ^a^ |
| Distant recurrence (n=101) | | | 8 (11.1) | 9 (31) | 0.04 ^a^ |
| Disease-free survival (n=101) | | | 57 (79.2) | 9 (31) | <0.01 ^a^ |
| Overall survival (n=101) | | | 62 (86.1) | 11 (37.9) | <0.01 ^a^ |

LaTME: Laparoscopic Total Mesorectal Excision. TaTME: Transanal Total Mesorectal Excision. IQR: Interquartile Range. BMI: Body Mass Index. ^a^ Pearson chi-square or Fisher’s Exact Test. ^b^ Mann-Whitney U test

**Table S5. Preop/Postop: LARS score, mITT**

| **Characteristics** | | **LaTME (n=33/50 [66%])**  Median (IQR) | **P value**  **Pre/Post**  **LaTME** | **TaTME**  **n=32/55 [58.2%]**  Median (IQR) | **P value**  **Pre/Post**  **TaTME** | **P value**  **Pre**  **LaTME/TaTME** | **P value**  **Post**  **LaTME/TaTME** |
| --- | --- | --- | --- | --- | --- | --- | --- |
| **Flatus incontinence** | Preop. | 4 (7) | 0.345^ | 0 (4) | 0.001^ | 0.161* | 0.101* |
|  | Postop. | 4 (7) |  | 7 (6) |  |  |  |
| **Liquid stool incontinence** | Preop. | 0 (3) | 0.248^ | 0 (0) | 0.000^ | 0.044* | 0.005* |
|  | Postop. | 0 (3) |  | 3 (3) |  |  |  |
| **Evacuation frequency** | Preop. | 0 (2) | 0.087^ | 0 (0) | 0.014^ | 0.294* | 0.801* |
|  | Postop. | 0 (3) |  | 0 (2) |  |  |  |
| **Fragmented evacuation** | Preop. | 0 (7) | 0.002^ | 0 (4) | 0.004^ | 0.750* | 0.535* |
|  | Postop. | 7 (7) |  | 4 (7) |  |  |  |
| **Evacuation urgency** | Preop. | 11 (16) | 0.059^ | 0 (11) | 0.001^ | 0.029* | 0.596* |
|  | Postop. | 16 (11) |  | 11 (5) |  |  |  |
| **LARS total** | Preop. | 19 (27) | 0.05^ | 4 (19) | 0.000^ | 0.048* | 0.454* |
|  | Postop. | 24 (19) |  | 27.5 (21) |  |  |  |

^Wilcoxon signed-rank test for paired samples. *Mann–Whitney U test for independent samples.

**Table S6. Preop/Postop LARS score, PP population**

| **Characteristics** | | **LaTME (n=30/40 [75%])**  **Median (IQR)** | **P value**  **Pre/Post**  **LaTME** | **TaTME**  **(n=32/54 [59.3 %])**  **Median (IQR)** | **P value**  **Pre/Post**  **TaTME** | **P value**  **Pre**  **LaTME/TaTME** | **P value**  **Post**  **LaTME/TaTME** |
| --- | --- | --- | --- | --- | --- | --- | --- |
| **Flatus incontinence** | Preop. | 4 (7) | 0.345^ | 0 (4) | 0.001^ | 0.161* | 0.101* |
|  | Postop. | 4 (7) |  | 7 (6) |  |  |  |
| **Liquid stool incontinence** | Preop. | 0 (3) | 0.248^ | 0 (0) | <0.001^ | 0.044* | 0.005* |
|  | Postop. | 0 (3) |  | 3 (3) |  |  |  |
| **Evacuation frequency** | Preop. | 0 (2) | 0.087^ | 0 (0) | 0.014^ | 0.294* | 0.801* |
|  | Postop. | 0 (3) |  | 0 (2) |  |  |  |
| **Fragmented evacuation** | Preop. | 0 (7) | 0.002^ | 0 (4) | 0.004^ | 0.750* | 0.535* |
|  | Postop. | 7 (7) |  | 4 (7) |  |  |  |
| **Evacuation urgency** | Preop. | 11 (16) | 0.059^ | 0 (11) | 0.001^ | 0.029* | 0.596* |
|  | Postop. | 16 (11) |  | 11 (5) |  |  |  |
| **LARS total** | Preop. | 19 (27) | 0.05^ | 4 (19) | <0.001^ | 0.048* | 0.454* |
|  | Postop. | 24 (19) |  | 27.5 (21) |  |  |  |

^Wilcoxon signed-rank test for paired samples. *Mann–Whitney U test for independent samples.

Table S7. Univariate analysis of Major LARS (Modified Intention-to-Treat)

|  | | **No /Minor LARS**  **(n=41)**  **Median (IQR)** | **Major LARS**  **(n=41)**  **Median (IQR)** | **P value** |
| --- | --- | --- | --- | --- |
| Sex (n, %) | Male | 32 (78) | 17 (70.8) | 0.56^ |
|  | Female | 9 (22) | 7 (29.2) |  |
| Age. Median (IQR) years | | 68 (17) | 57.5 (19) | 0.273* |
| BMI. Median (IQR), cm | | 27 (6.6) | 28.5 (10) | 0.9* |
| Height from anal verge, median (IQR), cm | | 9 (3) | 8 (3) | 0.335* |
| Treatment (n, %) | LaTME | 23 (56.1) | 10 (41.7) | 0.31^ |
|  | TaTME | 18 (43.9) | 14 (58.3) |  |
| Chemoradiotherapy (n, %) | | 28 (68.3) | 18 (75) | 0.778^ |
| Composite outcome of conversion surgery | | 2 (4.9) | 1 (4.2) | 0.99^ |
| Overall Morbidity (n, %) | | 14 (34.1) | 15 (62.5) | 0.039^ |
| Relevant morbidity (Cl-D > II) (n, %) | | 0 (0) | 3 (12.5) | 0.046^ |
| Anastomotic leak | | 4 (9.8) | 2 (8.3) | 0.99^ |
| CCI, median (IQR) | | 0 (29) | 0 (6) | 0.044* |
| **EORTC QLQ-C30 Post-operative Scores. Median (IQR)** | | | | |
|  | | **No /Minor LARS**  **(n=56)** | **Major LARS**  **(n=8)** | **P value** |
| **Global health status** | | 83.3 (16.7) | 83.3 (31.3) | < 0.001* |
| **Functional Scales**  Median (IQR) | Physical functioning | 100 (13.3) | 93.3 (23.3) | 0.007* |
|  | Role functioning | 100 (0) | 100 (58.3) | 0.008* |
|  | Emotional functioning | 100 (16.7) | 75 (56.3) | 0.131* |
|  | Cognitive functioning | 100 (16.7) | 100 (29.2) | 0.158* |
|  | Social functioning | 100 (33.3) | 83.3 (83.3) | 0.025* |
| **Symptom Scales**  Median (IQR) | Fatigue | 0 (22.2) | 22.2 (55.6) | 0.004 |
|  | Nausea and vomiting | 0 (0) | 0 (0) | 0.99 |
|  | Pain | 0 (12.5) | 0 (33.3) | 0.345 |
|  | Dyspnoea | 0 (0) | 0 (33.3) | 0.247 |
|  | Insomnia | 0 (33.3) | 0 (100) | 0.001 |
|  | Appetite loss | 0 (0) | 0 (0) | 0.127 |
|  | Constipation | 0 (33.3) | 0 (0) | 0.426 |
|  | Diarrhoea | 16.7 (66.7) | 33.3 (33.3) | <0.001 |
|  | Financial difficulties | 0 (25) | 0 (0) | 0.058 |
| **EORTC QLQ-CR29 Post-operative Scores. Median (IQR)** | | | | |
|  | | **No /Minor LARS**  **(n=62)** | **Major LARS**  **(n=10)** | **P value** |
| **Functional Scales**  Median (IQR) | Body Image | 100 (22.2) | 100 (22.2) | 0.9 |
|  | Sexual function (men) | 66.6 (33.3) | 66.7 (0) | 0.858 |
|  | Sexual function (women) | 100 (33.3) | 66.7 (-) | 0.536 |
| **Symptom Scales**  Median (IQR) | Urinary frequency | 16.7 (50) | 50 (45.8) | 0.982 |
|  | Blood and mucus with stool | 16.7 (50) | 25 (83.3) | 0.714 |
|  | Stool Frequency | 16.7 (29.2) | 25 (50) | <0.001 |
|  | Urinary Incontinence | 0 (33.3) | 0 (0) | 0.11 |
|  | Dysuria | 0 (0) | 0 (0) | 0.601 |
|  | Abdominal pain | 0 (25) | 0 (33.3) | 0.705 |
|  | Buttock pain | 0 (33.3) | 0 (25) | 0.290 |
|  | Bloated feeling | 0 (25) | 0 (0) | 0.537 |
|  | Dry mouth | 0 (33.3) | 0 (50) | 0.779 |
|  | Hair loss | 0 (0) | 0 (66.7) | 0.34 |
|  | Trouble with taste | 0 (0) | 0 (0) | 0.038 |
|  | Anxiety | 66.7 (33.3) | 66.7 (66.7) | 0.194 |
|  | Weight | 100 (0) | 83.3 (66.7) | 0.139 |
|  | Flatulence | 33.3 (66.7) | 33.3 (91.7) | <0.001 |
|  | Faecal Incontinence | 0 (66.7) | 33.3 (83.3) | <0.001 |
|  | Sore Skin | 0 (33.3) | 16.7 (91.7) | 0.013 |
|  | Embarrassment by bowel movement | 0 (33.3) | 16.7 (58.3) | <0.001 |
|  | Impotence | 33.3 (66.7) | 0 (66.7) | 0.963 |
|  | Dyspareunia | 0 (0) | 0 (0) | 0.491 |
|  |  |  |  |  |

LARS : Low Anterior Resection Syndrome. BMI: Body Mass Index. LaTME: Laparoscopic Total Mesorectal Excision. TaTME: Transanal Total Mesorectal Excision. IQR: Interquartile Range. *Independent-Samples Mann-Whitney U Test. ^ Pearson's Χ2 test or Fisher's exact test. ^$^ Independent-Samples T-Test

**Supplementary Figures**

**Figure S2**

**Figure S3**

**Figure S4**

**Figure S5**
