# Supplementary material for: Quality of life, bowel dysfunction, and long-term oncological outcomes after transanal versus laparoscopic total mesorectal excision for mid- and low- rectal cancer (Ta-LaTME study): multicentre randomized open-label trial
Source: BJS Open. 2026 Aug 4;10(4):zrag045. doi: 10.1093/bjsopen/zrag045 (PMC13436816; doi:10.1093/bjsopen/zrag045)
Supplement: Supplementary file 1 [file zrag045_supplementary_data.zip › Table_S1_REV1_Q1_Q3_BJSOpen.docx]

Table S1. Patients’ characteristics with valid and no valid questionnaires EORTC QLQ-C30/CR29

| **Baseline characteristics**  **All patients (n=105)** | | | **Valid questionnaires (n=67, 63.8%)** | **No valid questionnaires (n=38, 36.2%)** | **P value** |
| --- | --- | --- | --- | --- | --- |
| Sex | | Women | 20 (29.9%) | 10 (26.3) | 0.82^a^ |
|  |  | Men | 47 (70.1) | 28 (73.1) |  |
| Age, Median (Q1, Q3), years | | | 65 (54.8, 72.3) | 72.5 (61.8, 79.3) | 0.01^b^ |
| BMI, Median (Q1, Q3) | | | 27 (24.4, 30.3) | 25.6 (23.5, 28.9) | 0.13 ^b^ |
| ASA score | | I | 3 (4.5) | 2 (5.3) | <0.01 |
|  |  | II | 47 (70.1) | 14 (36.8) |  |
|  |  | III | 17 (24.4) | 22 (57.9) |  |
| Neoadjuvant treatment (n=65) | | | 48 (71.6) | 17 (44.7) | 0.01 ^a^ |
| Clinical TNM | | T1 | 1 (1.5) | 2 (5.3) | 0.49 ^a^ |
| T  N | | T2 | 10 (14.9) | 8 (21.1) |  |
|  |  | T3ab | 30 (44.8) | 17 (44.7) |  |
|  |  | T3cd | 26 (38.8) | 11 (28.9) |  |
|  |  | N0 | 29 (43.3) | 22 (57.9) | 0.33 ^a^ |
|  |  | N1 | 28 (41.8) | 11 (28.9) |  |
|  |  | N2 | 10 (14.9) | 5 (13.2) |  |
| Tumour height, Median (Q1, Q3), cm | | | 9 (7, 10) | 8 (7, 10) | 0.58 ^b^ |
| LaTME/TaTME | LaTME | | 34 (50.7) | 16 (42.1) | 0.42 ^a^ |
|  | TaTME | | 33 (49.3) | 22 (57.9) |  |
| Defunctioning stoma | Ileostomy | | 64 (95.5) | 33 (86.8) | 0.21 ^a^ |
|  | Colostomy | | 1 (1.5) | 3 (7.9) |  |
|  | None | | 2 (3) | 2 (5.3) |  |
| Overall morbidity | | | 21 (31.3) | 18 (47.3) | 0.09 ^a^ |
| Relevant morbidity (Cl–D > II) | | | 3 (4.5) | 7 (18.4) | 0.03 ^a^ |
| Anastomotic leak | | | 1 (2.6) | 6 (9) | 0.21 ^a^ |
| Time to stoma closure, Median (Q1, Q3), months | | | 13 (13, 14) | 13 (13, 13.5) | 0.22 ^b^ |
| Follow-up time, Median (Q1, Q3), months | | | 61.5 (50, 79.5) | 43 (30, 66) | <0.01 ^b^ |
| Local recurrence (n=101) | | | 1 (1.5) | 4 (11.8) | 0.04 ^a^ |
| Distant recurrence (n=101) | | | 5 (7.5) | 12 (35.3) | 0.01 ^a^ |
| Disease-free survival (n=101) | | | 58 (86.6) | 8 (23.5) | < 0.01 ^a^ |
| Overall survival (n=101) | | | 64 (95.5) | 9 (26.5) | < 0.01 ^a^ |

LaTME: Laparoscopic Total Mesorectal Excision. TaTME: Transanal Total Mesorectal Excision. Categorical variables are expressed as n (%). Quantitative variables are expressed as median (Q1, Q3), where Q1 and Q3 denote the first and third quartiles, respectively. BMI: Body Mass Index. a Pearson chi-square or Fisher’s exact test. b Mann-Whitney U test
