# Supplementary material for: Quality of life, bowel dysfunction, and long-term oncological outcomes after transanal versus laparoscopic total mesorectal excision for mid- and low- rectal cancer (Ta-LaTME study): multicentre randomized open-label trial
Source: BJS Open. 2026 Aug 4;10(4):zrag045. doi: 10.1093/bjsopen/zrag045 (PMC13436816; doi:10.1093/bjsopen/zrag045)
Supplement: Supplementary file 1 [file zrag045_supplementary_data.zip › Table_S2_REV1_Q1_Q3_BJSOpen.docx]

Table S2. Preoperative and postoperative EORTC QLQ-C30 results (PP population)

| **Characteristics** | | **LaTME**  **(n=40)**  **Median (Q1, Q3)** | **CID- LaTME** | **ES- LaTME** | **SRM- LaTME** | **P value**  **Pre/Post**  **LaTME** | **TaTME**  **(n=54)**  **Median (Q1, Q3)** | **CID- TaTME** | **ES- TaTME** | **SRM- TaTME** | **P value**  **Pre/Post**  **TaTME** | **P value**  **Pre/Post**  **LaTME / TaTME** |
| --- | --- | --- | --- | --- | --- | --- | --- | --- | --- | --- | --- | --- |
| **Global health status** | Preop. | 79.2 (56.2, 100.0) | -4.30 | -0.19 | -0.18 | 0.489^ | 83.3 (66.7, 100.0) | 7.83 | 0.45 | 0.29 | 0.108^ | 0.336* |
|  | Postop. | 83.3 (66.7, 93.8) |  |  |  |  | 83.3 (66.7, 83.3) |  |  |  |  | 0.236* |
| **Functional Scales** | | | | | | | | | | | | |
| Physical functioning | Preop. | 100.0 (86.7, 100.0) | -3.66 | -0.28 | -0.25 | 0.194^ | 100.0 (96.7, 100.0) | 1.21 | 0.07 | 0.07 | 0.314^ | 0.172* |
|  | Postop. | 100.0 (93.3, 100.0) |  |  |  |  | 100.0 (86.7, 100.0) |  |  |  |  | 0.117* |
| Role functioning | Preop. | 100.0 (100.0, 100.0) | 3.23 | 0.22 | 0.12 | 0.526^ | 100.0 (100.0, 100.0) | 2.02 | 0.13 | 0.07 | 0.812^ | 0.629* |
|  | Postop. | 100.0 (100.0, 100.0) |  |  |  |  | 100.0 (100.0, 100.0) |  |  |  |  | 0.700* |
| Emotional functioning | Preop. | 83.3 (50.0, 100.0) | -15.59 | -0.56 | -0.49 | 0.017^ | 83.3 (75.0, 83.3) | -8.59 | -0.49 | -0.32 | 0.052^ | 0.978* |
|  | Postop. | 100.0 (83.3, 100.0) |  |  |  |  | 100.0 (83.3, 100.0) |  |  |  |  | 0.393* |
| Cognitive functioning | Preop. | 100.0 (83.3, 100.0) | -6.45 | -0.33 | -0.36 | 0.055^ | 100.0 (91.7, 100.0) | 5.05 | 0.38 | 0.26 | 0.157^ | 0.440* |
|  | Postop. | 100.0 (100.0, 100.0) |  |  |  |  | 100.0 (75.0, 100.0) |  |  |  |  | 0.017* |
| Social functioning | Preop. | 100.0 (83.3, 100.0) | 5.91 | 0.26 | 0.19 | 0.506^ | 100.0 (83.3, 100.0) | 1.01 | 0.04 | 0.03 | 0.981^ | 0.665* |
|  | Postop. | 100.0 (66.7, 100.0) |  |  |  |  | 100.0 (66.7, 100.0) |  |  |  |  | 0.704* |
| **Symptom Scales** | | | | | | | | | | | | |
| Fatigue | Preop. | 22.2 (0.0, 33.3) | 8.96 | 0.40 | 0.35 | 0.042^ | 0.0 (0.0, 27.8) | -3.70 | -0.18 | -0.12 | 0.438^ | 0.070* |
|  | Postop. | 0.0 (0.0, 22.2) |  |  |  |  | 11.1 (0.0, 27.8) |  |  |  |  | 0.349* |
| Nausea and vomiting | Preop. | 0.0 (0.0, 0.0) | 2.69 | 0.22 | 0.25 | 0.180^ | 0.0 (0.0, 0.0) | -1.01 | -0.21 | -0.09 | 0.680^ | 0.762* |
|  | Postop. | 0.0 (0.0, 0.0) |  |  |  |  | 0.0 (0.0, 0.0) |  |  |  |  | 0.570* |
| Pain | Preop. | 0.0 (0.0, 33.3) | 8.60 | 0.70 | 0.25 | 0.124^ | 0.0 (0.0, 8.3) | -5.05 | -0.38 | -0.24 | 0.217^ | 0.031* |
|  | Postop. | 0.0 (0.0, 0.0) |  |  |  |  | 0.0 (0.0, 16.7) |  |  |  |  | 0.500* |
| Dyspnoea | Preop. | 0.0 (0.0, 0.0) | 2.98 | 0.15 | 0.12 | 0.534^ | 0.0 (0.0, 0.0) | -1.01 | -0.10 | -0.05 | 1.000^ | 0.118* |
|  | Postop. | 0.0 (0.0, 0.0) |  |  |  |  | 0.0 (0.0, 0.0) |  |  |  |  | 0.198* |
| Insomnia | Preop. | 0.0 (0.0, 33.3) | 6.45 | 0.20 | 0.21 | 0.194^ | 0.0 (0.0, 33.3) | 8.08 | 0.24 | 0.19 | 0.374^ | 0.907* |
|  | Postop. | 0.0 (0.0, 33.3) |  |  |  |  | 0.0 (0.0, 0.0) |  |  |  |  | 0.461* |
| Appetite loss | Preop. | 0.0 (0.0, 33.3) | 6.45 | 0.32 | 0.30 | 0.196^ | 0.0 (0.0, 0.0) | -5.05 | -0.3 | -0.2 | 0.473^ | 0.099* |
|  | Postop. | 0.0 (0.0, 0.0) |  |  |  |  | 0.0 (0.0, 16.7) |  |  |  |  | 0.137* |
| Constipation | Preop. | 0.0 (0.0, 33.3) | 1.08 | 0.06 | 0.04 | 0.924^ | 0.0 (0.0, 0.0) | -14.14 | -0.53 | -0.32 | 0.077^ | 0.037* |
|  | Postop. | 0.0 (0.0, 33.3) |  |  |  |  | 0.0 (0.0, 50.0) |  |  |  |  | 0.792* |
| Diarrhoea | Preop. | 0.0 (0.0, 66.7) | 1.08 | 0.03 | 0.03 | 0.853^ | 0.0 (0.0, 33.3) | -17.17 | -0.52 | -0.34 | 0.068^ | 0.710* |
|  | Postop. | 0.0 (0.0, 33.3) |  |  |  |  | 33.3 (0.0, 83.3) |  |  |  |  | 0.252* |
| Financial difficulties | Preop. | 0.0 (0.0, 33.3) | 4.30 | 0.13 | 0.13 | 0.556^ | 0.0 (0.0, 50.0) | 8.08 | 0.23 | 0.23 | 0.152^ | 0.828* |
|  | Postop. | 0.0 (0.0, 0.0) |  |  |  |  | 0.0 (0.0, 16.7) |  |  |  |  | 0.935* |

Data are expressed as median (Q1, Q3), where Q1 and Q3 denote the first and third quartiles, respectively. ^ Wilcoxon signed-rank test (within-group comparisons). * Mann–Whitney U test (between-group comparisons). CID = clinically important difference; ES = effect size; SRM = standardized response mean.
