# Supplementary material for: Quality of life, bowel dysfunction, and long-term oncological outcomes after transanal versus laparoscopic total mesorectal excision for mid- and low- rectal cancer (Ta-LaTME study): multicentre randomized open-label trial
Source: BJS Open. 2026 Aug 4;10(4):zrag045. doi: 10.1093/bjsopen/zrag045 (PMC13436816; doi:10.1093/bjsopen/zrag045)
Supplement: Supplementary file 1 [file zrag045_supplementary_data.zip › Table_S3_REV1_Q1_Q3_BJSOpen.docx]

Table S3. Preoperative and postoperative EORTC QLQ-CR29 results (PP population)

| **Characteristics** | | **LaTME (n=40) Median (Q1, Q3)** | **CID- LaTME** | **ES- LaTME** | **SRM- LaTME** | **P value Pre/Post LaTME** | **TaTME (n=54) Median (Q1, Q3)** | **CID- TaTME** | **ES- TaTME** | **SRM- TaTME** | **P value Pre/Post TaTME** | **P value Pre/Post LaTME/TaTME** |
| --- | --- | --- | --- | --- | --- | --- | --- | --- | --- | --- | --- | --- |
| **Functional Scales** | | | | | | | | | | | | |
| Body Image | Preop. | 100 (75, 100) | -3.58 | -0.22 | -0.12 | 0.233^ | 100 (88.9, 100) | 1.16 | -0.13 | -0.13 | 0.548^ | 0.297* |
|  | Postop. | 100 (100, 100) |  |  |  |  | 100 (88.9, 100) |  |  |  |  | 0.486* |
| Sexual function (men) | Preop. | 66.7 (66.7, 100) | -2.07 | 0 | 0 | 0.672^ | 66.7 (66.7, 100) | 0.51 | 0.07 | 0.05 | 0.618^ | 0.305* |
|  | Postop. | 66.7 (33.3, 100) |  |  |  |  | 66.7 (66.7, 100) |  |  |  |  | 0.364* |
| Sexual function (women) | Preop. | 100 (66.7, 100) | 3.91 | 0.17 | 0.20 | 1.000^ | 66.7 (66.7, 100) | -19.05 | -0.83 | -1.07 | 0.059^ | 0.252* |
|  | Postop. | 83.3 (66.7, 100) |  |  |  |  | 100 (66.7, 100) |  |  |  |  | 0.475* |
| **Symptom Scales** | | | | | | | | | | | | |
| Urinary frequency | Preop. | 33.3 (0, 54.2) | 20.43 | 0.70 | 0.54 | 0.006^ | 0 (0, 50) | 2.02 | 0.07 | 0.05 | 0.786^ | 0.104* |
|  | Postop. | 0 (0, 33.3) |  |  |  |  | 16.7 (0, 33.3) |  |  |  |  | 0.176* |
| Blood and mucus with stool | Preop. | 16.7 (0, 50) | 29.03 | 0.96 | 1.00 | <0.001^ | 33.3 (0, 50) | 23.74 | 0.85 | 0.75 | 0.001^ | 0.792* |
|  | Postop. | 0 (0, 0) |  |  |  |  | 0 (0, 0) |  |  |  |  | 0.053* |
| Stool frequency | Preop. | 16.7 (0, 33.3) | -5.75 | -0.31 | -0.18 | 0.581^ | 8.3 (0, 16.7) | -21.43 | -0.88 | -0.52 | 0.017^ | 0.484* |
|  | Postop. | 16.7 (0, 33.3) |  |  |  |  | 33.3 (16.7, 62.5) |  |  |  |  | 0.028* |
| Urinary incontinence | Preop. | 0 (0, 0) | -1.08 | -0.05 | -0.04 | 0.862^ | 0 (0, 0) | -8.08 | -0.42 | -0.39 | 0.029^ | 0.795* |
|  | Postop. | 0 (0, 0) |  |  |  |  | 0 (0, 33.3) |  |  |  |  | 0.197* |
| Dysuria | Preop. | 0 (0, 0) | 0 | 0 | 0 | 1.000^ | 0 (0, 0) | -5.05 | --- | -0.27 | 0.102^ | 0.141* |
|  | Postop. | 0 (0, 0) |  |  |  |  | 0 (0, 0) |  |  |  |  | 0.675* |
| Abdominal pain | Preop. | 0 (0, 33.3) | 6.45 | 0.24 | 0.20 | 0.294^ | 0 (0, 33.3) | 3.03 | 0.11 | 0.09 | 0.557^ | 0.447* |
|  | Postop. | 0 (0, 33.3) |  |  |  |  | 0 (0, 16.7) |  |  |  |  | 0.936* |
| Buttock pain | Preop. | 0 (0, 33.3) | 1.08 | 0.04 | 0.03 | 0.958^ | 0 (0, 0) | -9.09 | -0.62 | -0.27 | 0.146^ | 0.090* |
|  | Postop. | 0 (0, 33.3) |  |  |  |  | 0 (0, 33.3) |  |  |  |  | 0.896* |
| Bloated feeling | Preop. | 0 (0, 33.3) | 7.53 | 0.30 | 0.25 | 0.185^ | 0 (0, 33.3) | 1.01 | 0.03 | 0.03 | 0.936^ | 0.861* |
|  | Postop. | 0 (0, 0) |  |  |  |  | 0 (0, 33.3) |  |  |  |  | 0.340* |
| Dry mouth | Preop. | 0 (0, 41.7) | 15.05 | 0.44 | 0.41 | 0.038^ | 0 (0, 33.3) | -3.03 | -0.11 | -0.08 | 0.675^ | 0.186* |
|  | Postop. | 0 (0, 33.3) |  |  |  |  | 0 (0, 33.3) |  |  |  |  | 0.423* |
| Hair loss | Preop. | 0 (0, 0) | 2.15 | 0.08 | 0.06 | 0.762^ | 0 (0, 33.3) | 6.06 | 0.24 | 0.25 | 0.403^ | 0.278* |
|  | Postop. | 0 (0, 0) |  |  |  |  | 0 (0, 0) |  |  |  |  | 0.915* |
| Trouble with taste | Preop. | 0 (0, 8.3) | 8.60 | 0.31 | 0.25 | 0.207^ | 0 (0, 16.7) | 3.03 | 0.09 | 0.08 | 0.776^ | 0.807* |
|  | Postop. | 0 (0, 0) |  |  |  |  | 0 (0, 0) |  |  |  |  | 0.239* |
| Anxiety | Preop. | 66.7 (33.3, 100) | -18.28 | -0.54 | -0.48 | 0.008^ | 66.7 (33.3, 100) | -10.23 | -0.38 | -0.29 | 0.277^ | 0.285* |
|  | Postop. | 66.7 (66.7, 100) |  |  |  |  | 66.7 (66.7, 100) |  |  |  |  | 0.960* |
| Weight | Preop. | 100 (66.7, 100) | -4.30 | -0.17 | -0.14 | 0.417^ | 100 (66.7, 100) | -1.92 | -0.08 | -0.07 | 0.892^ | 0.993* |
|  | Postop. | 100 (66.7, 100) |  |  |  |  | 100 (10, 100) |  |  |  |  | 0.669* |
| Flatulence | Preop. | 16.7 (0, 66.7) | -2.95 | -0.08 | -0.06 | 0.917^ | 0 (0, 33.3) | -27.08 | -0.89 | -0.60 | 0.008^ | 0.132* |
|  | Postop. | 33.3 (0, 33.3) |  |  |  |  | 33.3 (0, 66.7) |  |  |  |  | 0.143* |
| Faecal incontinence | Preop. | 0 (0, 33.3) | 0.08 | -0.10 | -0.06 | 0.676^ | 0 (0, 0) | -29.91 | -1.03 | -0.77 | 0.002^ | 0.061* |
|  | Postop. | 0 (0, 33.3) |  |  |  |  | 33.3 (0, 66.7) |  |  |  |  | 0.010* |
| Sore skin | Preop. | 0 (0, 33.3) | -7.17 | -0.48 | -0.27 | 0.188^ | 0 (0, 0) | -28.87 | -2.08 | -0.61 | 0.006^ | 0.024* |
|  | Postop. | 0 (0, 33.3) |  |  |  |  | 0 (0, 66.7) |  |  |  |  | 0.341* |
| Embarrassment by bowel movement | Preop. | 0 (0, 8.3) | -6.41 | -0.42 | -0.18 | 0.445^ | 0 (0, 0) | -13.10 | -0.69 | -0.53 | 0.013^ | 0.049* |
|  | Postop. | 0 (0, 33.3) |  |  |  |  | 0 (0, 33.3) |  |  |  |  | 0.330* |
| Stoma care problems | Preop. | --- | --- | --- | --- | --- | --- | --- | --- | --- | --- | --- |
|  | Postop. | --- |  |  |  |  | --- |  |  |  |  | --- |
| Impotence | Preop. | 33.3 (0, 66.7) | 7.84 | 0.21 | 0.18 | 0.463^ | 0 (0, 16.7) | -21.95 | -0.81 | -0.67 | 0.011^ | 0.064* |
|  | Postop. | 33.3 (0, 58.3) |  |  |  |  | 33.3 (0, 66.7) |  |  |  |  | 0.588* |
| Dyspareunia | Preop. | 0 (0, 33.3) | -3.81 | -0.68 | -0.41 | 0.317^ | 0 (0, 66.7) | 14.29 | 0.34 | 0.38 | 0.317^ | 0.805* |
|  | Postop. | 0 (0, 33.3) |  |  |  |  | 0 (0, 0) |  |  |  |  | 0.669* |

Data are expressed as median (Q1, Q3), where Q1 and Q3 denote the first and third quartiles, respectively. ^ Wilcoxon signed-rank test (within-group comparisons). * Mann–Whitney U test (between-group comparisons). CID = clinically important difference; ES = effect size; SRM = standardized response mean.
