# Supplementary material for: Quality of life, bowel dysfunction, and long-term oncological outcomes after transanal versus laparoscopic total mesorectal excision for mid- and low- rectal cancer (Ta-LaTME study): multicentre randomized open-label trial
Source: BJS Open. 2026 Aug 4;10(4):zrag045. doi: 10.1093/bjsopen/zrag045 (PMC13436816; doi:10.1093/bjsopen/zrag045)
Supplement: Supplementary file 1 [file zrag045_supplementary_data.zip › Table_S4_REV1_Q1_Q3_BJSOpen.docx]

Table S4. Patients’ characteristics with valid and no valid questionnaires LARS

| **Baseline characteristics**  **All patients (n=105)** | | | **Valid questionnaires (n=72, 68.6%)** | **No valid questionnaires (n=33, 31.4%)** | **P value** |
| --- | --- | --- | --- | --- | --- |
| Sex | | Women | 20 (27.8) | 10 (30.3) | 0.82 ^a^ |
|  |  | Men | 52 (72.2) | 23 (69.7) |  |
| Age, Median (Q1, Q3), years | | | 66.5 (56, 73) | 72 (65, 80) | 0.07 ^b^ |
| BMI, Median (Q1, Q3) | | | 26.7 (24.1, 29.8) | 25.7 (23.2, 28.6) | 0.59 ^b^ |
| ASA score | | I | 4 (5.6) | 1 (3) | 0.01 ^a^ |
|  |  | II | 48 (66.7) | 13 (39.4) |  |
|  |  | III | 20 (27.8) | 19 (57.6) |  |
| Neoadjuvant treatment (n=65) | | | 51 (70.8) | 14 (42.4) | 0.01 ^a^ |
| Clinical TNM | | T1 | 1 (1.4) | 2 (6.1) | 0.43 ^a^ |
| T  N | | T2 | 12 (16.7) | 6 (18.2) |  |
|  |  | T3ab | 31 (43.1) | 16 (48.5) |  |
|  |  | T3cd | 28 (38.9) | 9 (27.3) |  |
|  |  | N0 | 30 (41.7) | 21 (63.6) | 0.1 ^a^ |
|  |  | N1 | 31 (43.1) | 8 (24.2) |  |
|  |  | N2 | 11 (15.3) | 4 (12.1) |  |
| Tumour height, Median (Q1, Q3), cm | | | 9 (7, 10) | 8 (6, 9) | 0.46 ^b^ |
| LaTME/TaTME | LaTME | | 33 (45.8) | 17 (51.5) | 0.68 ^a^ |
|  | TaTME | | 39 (54.2) | 16 (48.5) |  |
| Defunctioning stoma | Ileostomy | | 68 (94.4) | 29 (87.9) | 0.16 ^a^ |
|  | Colostomy | | 1 (1.4) | 3 (9.1) |  |
|  | None | | 3 (4.2) | 1 (3) |  |
| Overall morbidity | | | 27 (37.5) | 12 (36.4) | 0.83 ^a^ |
| Relevant morbidity (Cl–D > II) | | | 4 (5.6) | 6 (18.2) | 0.07 ^a^ |
| Anastomotic leak | | | 6 (8.3) | 1 (3) | 0.43 ^a^ |
| Time to stoma closure, Median (Q1, Q3), months | | | 13 (13, 14) | 13 (13, 14.5) | 0.33 ^b^ |
| Follow-up time, Median (Q1, Q3), months | | | 60 (50.5, 73) | 42 (35, 64) | <0.01 ^b^ |
| Local recurrence (n=101) | | | 2 (2.8) | 3 (10.3) | 0.14 ^a^ |
| Distant recurrence (n=101) | | | 8 (11.1) | 9 (31) | 0.04 ^a^ |
| Disease-free survival (n=101) | | | 57 (79.2) | 9 (31) | <0.01 ^a^ |
| Overall survival (n=101) | | | 62 (86.1) | 11 (37.9) | <0.01 ^a^ |

LaTME: Laparoscopic Total Mesorectal Excision. TaTME: Transanal Total Mesorectal Excision. Categorical variables are expressed as n (%). Quantitative variables are expressed as median (Q1, Q3), where Q1 and Q3 denote the first and third quartiles, respectively. BMI: Body Mass Index. a Pearson chi-square or Fisher’s exact test. b Mann-Whitney U test
