# Supplementary material for: Quality of life, bowel dysfunction, and long-term oncological outcomes after transanal versus laparoscopic total mesorectal excision for mid- and low- rectal cancer (Ta-LaTME study): multicentre randomized open-label trial
Source: BJS Open. 2026 Aug 4;10(4):zrag045. doi: 10.1093/bjsopen/zrag045 (PMC13436816; doi:10.1093/bjsopen/zrag045)
Supplement: Supplementary file 1 [file zrag045_supplementary_data.zip › Table_S5_REV1_Q1_Q3_BJSOpen.docx]

Table S5. Preoperative and postoperative LARS score items (mITT population)

| **Characteristics** | | **LaTME (n=33/50 [66%])**  Median (Q1, Q3) | **P value**  **Pre/Post**  **LaTME** | **TaTME**  **(n=32/55 [58.2%])**  Median (Q1, Q3) | **P value**  **Pre/Post**  **TaTME** | **P value**  **Pre**  **LaTME/TaTME** | **P value**  **Post**  **LaTME/TaTME** |
| --- | --- | --- | --- | --- | --- | --- | --- |
| **Incontinence for flatus** | Preop. | 4 (0, 7) | 0.345^ | 0 (0, 4) | 0.001^ | 0.161* | 0.101* |
|  | Postop. | 4 (0, 7) |  | 7 (1, 7) |  |  |  |
| **Incontinence for liquid stools** | Preop. | 0 (0, 3) | 0.248^ | 0 (0, 0) | <0.001^ | 0.044* | 0.005* |
|  | Postop. | 0 (0, 3) |  | 3 (0, 3) |  |  |  |
| **Frequency of bowel movements** | Preop. | 0 (0, 2) | 0.087^ | 0 (0, 0) | 0.014^ | 0.294* | 0.801* |
|  | Postop. | 0 (0, 3) |  | 0 (0, 2) |  |  |  |
| **Clustering of stools** | Preop. | 0 (0, 7) | 0.002^ | 0 (0, 4) | 0.004^ | 0.750* | 0.535* |
|  | Postop. | 7 (0, 7) |  | 4 (0, 7) |  |  |  |
| **Urgency** | Preop. | 11 (0, 16) | 0.059^ | 0 (0, 11) | 0.001^ | 0.029* | 0.596* |
|  | Postop. | 13.5 (8.3, 16) |  | 11 (11, 16) |  |  |  |
| **Total LARS score** | Preop. | 17 (0, 26) | 0.050^ | 5.5 (0, 15.8) | <0.001^ | 0.048* | 0.454* |
|  | Postop. | 24 (13.5, 32) |  | 27.5 (14.3, 34.5) |  |  |  |

Data are expressed as median (Q1, Q3), where Q1 and Q3 denote the first and third quartiles, respectively. ^ Wilcoxon signed-rank test for paired samples. * Mann-Whitney U test for independent samples.
