# Supplementary material for: Quality of life, bowel dysfunction, and long-term oncological outcomes after transanal versus laparoscopic total mesorectal excision for mid- and low- rectal cancer (Ta-LaTME study): multicentre randomized open-label trial
Source: BJS Open. 2026 Aug 4;10(4):zrag045. doi: 10.1093/bjsopen/zrag045 (PMC13436816; doi:10.1093/bjsopen/zrag045)
Supplement: Supplementary file 1 [file zrag045_supplementary_data.zip › Table_S7_REV1_Q1_Q3_BJSOpen.docx]

Table S7. Univariate analysis of major LARS (modified intention-to-treat population)

|  | | **No/Minor LARS**  **(n=41)** | **Major LARS**  **(n=24)** | **P value** |
| --- | --- | --- | --- | --- |
| Sex (n, %) | Male | 32 (78.0) | 17 (70.8) | 0.560^ |
|  | Female | 9 (22.0) | 7 (29.2) |  |
| Age, Median (Q1, Q3), years | | 68 (60.8, 73.8) | 63 (53.5, 71.8) | 0.273* |
| BMI, Median (Q1, Q3) | | 27 (24.5, 29.7) | 26.8 (24.2, 29.8) | 0.900* |
| Height from anal verge, Median (Q1, Q3), cm | | 9 (7, 10) | 9 (7, 10) | 0.335* |
| Treatment (n, %) | LaTME | 23 (56.1) | 10 (41.7) | 0.310^ |
|  | TaTME | 18 (43.9) | 14 (58.3) |  |
| Chemoradiotherapy (n, %) | | 28 (68.3) | 18 (75.0) | 0.778^ |
| Composite outcome of conversion surgery | | 2 (4.9) | 1 (4.2) | 0.990^ |
| Overall morbidity (n, %) | | 14 (34.1) | 15 (62.5) | 0.039^ |
| Relevant morbidity (Cl-D > II) (n, %) | | 0 (0) | 3 (12.5) | 0.046^ |
| Anastomotic leak | | 4 (9.8) | 2 (8.3) | 0.990^ |
| CCI, Median (Q1, Q3) | | 0 (0, 23) | 10.5 (0, 30) | 0.044* |
| **EORTC QLQ-C30 postoperative scores. Median (Q1, Q3)** | | | | |
|  | | **No/Minor LARS**  **(n=41)** | **Major LARS**  **(n=24)** | **P value** |
| **Global health status** | | 83.3 (83.3, 100) | 66.7 (58.3, 83.3) | <0.001* |
| **Functional Scales**  Median (Q1, Q3) | Physical functioning | 100 (96.7, 100) | 93.3 (86.7, 100) | 0.007* |
|  | Role functioning | 100 (100, 100) | 100 (83.3, 100) | 0.008* |
|  | Emotional functioning | 100 (91.7, 100) | 100 (83.3, 100) | 0.131* |
|  | Cognitive functioning | 100 (100, 100) | 100 (83.3, 100) | 0.158* |
|  | Social functioning | 100 (100, 100) | 100 (66.7, 100) | 0.025* |
| **Symptom Scales**  Median (Q1, Q3) | Fatigue | 0 (0, 11.1) | 22.2 (0, 33.3) | 0.004* |
|  | Nausea and vomiting | 0 (0, 0) | 0 (0, 0) | 0.990* |
|  | Pain | 0 (0, 0) | 0 (0, 0) | 0.345* |
|  | Dyspnoea | 0 (0, 0) | 0 (0, 0) | 0.247* |
|  | Insomnia | 0 (0, 0) | 0 (0, 33.3) | 0.001* |
|  | Appetite loss | 0 (0, 0) | 0 (0, 0) | 0.127* |
|  | Constipation | 0 (0, 33.3) | 0 (0, 66.7) | 0.426* |
|  | Diarrhoea | 0 (0, 33.3) | 66.7 (33.3, 100) | <0.001* |
|  | Financial difficulties | 0 (0, 0) | 0 (0, 33.3) | 0.058* |
| **EORTC QLQ-CR29 postoperative scores. Median (Q1, Q3)** | | | | |
|  | | **No /Minor LARS**  **(n=41)** | **Major LARS**  **(n=24)** | **P value** |
| **Functional Scales**  Median (Q1, Q3) | Body Image | 100 (100, 100) | 100 (88.6, 100) | 0.900* |
|  | Sexual function (men) | 66.7 (33.3, 100) | 66.7 (50, 100) | 0.858* |
|  | Sexual function (women) | 66.7 (66.7, 66.7) | 100 (66.7, 100) | 0.536* |
| **Symptom Scales**  Median (Q1, Q3) | Urinary frequency | 0 (0, 33.3) | 0 (0, 33.3) | 0.982* |
|  | Blood and mucus with stool | 0 (0, 0) | 0 (0, 0) | 0.714* |
|  | Stool frequency | 16.7 (0, 33.3) | 50 (20.8, 66.7) | <0.001* |
|  | Urinary Incontinence | 0 (0, 33.3) | 0 (0, 33.3) | 0.110* |
|  | Dysuria | 0 (0, 0) | 0 (0, 0) | 0.601* |
|  | Abdominal pain | 0 (0, 16.7) | 0 (0, 0) | 0.705* |
|  | Buttock pain | 0 (0, 16.7) | 0 (0, 33.3) | 0.290* |
|  | Bloated feeling | 0 (0, 0) | 0 (0, 25) | 0.537* |
|  | Dry mouth | 0 (0, 33.3) | 0 (0, 33.3) | 0.779* |
|  | Hair loss | 0 (0, 0) | 0 (0, 0) | 0.340* |
|  | Trouble with taste | 0 (0, 0) | 0 (0, 0) | 0.038* |
|  | Anxiety | 100 (66.7, 100) | 66.7 (33.3, 100) | 0.194* |
|  | Weight | 100 (100, 100) | 100 (100, 100) | 0.139* |
|  | Flatulence | 33.3 (0, 33.3) | 66.6 (33.3, 100) | <0.001* |
|  | Faecal Incontinence | 0 (0, 33.3) | 66.7 (33.3, 66.7) | <0.001* |
|  | Sore Skin | 0 (0, 0) | 33.3 (0, 66.7) | 0.013* |
|  | Embarrassment by bowel movement | 0 (0, 0) | 33.3 (0, 58.3) | <0.001* |
|  | Impotence | 33.3 (0, 66.7) | 33.3 (0, 83.3) | 0.963* |
|  | Dyspareunia | 0 (0, 0) | 0 (0, 41.7) | 0.491* |
|  |  |  |  |  |

LARS: Low Anterior Resection Syndrome. BMI: Body Mass Index. LaTME: Laparoscopic Total Mesorectal Excision. TaTME: Transanal Total Mesorectal Excision. Quantitative variables are expressed as median (Q1, Q3), where Q1 and Q3 denote the first and third quartiles, respectively. * Independent-samples Mann-Whitney U test. ^ Pearson's chi-square test or Fisher's exact test.
